# Negative pressure wound therapy in patients with wounds healing by secondary intention: a systematic review and meta-analysis of randomised controlled trials

**DOI:** 10.1186/s13643-020-01476-6

**Published:** 2020-10-10

**Authors:** Yvonne Zens, Michael Barth, Heiner C. Bucher, Katrin Dreck, Moritz Felsch, Wolfram Groß, Thomas Jaschinski, Heike Kölsch, Mandy Kromp, Inga Overesch, Stefan Sauerland, Sven Gregor

**Affiliations:** 1 Institute for Quality and Efficiency in Health Care (IQWiG), Im Mediapark 8, 50670 Cologne, Germany; 2 Basel Institute for Clinical Epidemiology & Biostatistics, University Hospital Basel and University of Basel, Basel, Switzerland; 3 Düsseldorf, Germany

**Keywords:** Negative-pressure wound therapy, Wound healing, Benefit assessment, Systematic review, Publication bias

## Abstract

**Background:**

Negative pressure wound therapy (NPWT) is a widely used method of wound treatment. We performed a systematic review of randomised controlled trials (RCTs) comparing the patient-relevant benefits and harms of NPWT with standard wound therapy (SWT) in patients with wounds healing by secondary intention.

**Methods:**

We searched for RCTs in MEDLINE, Embase, the Cochrane Central Register of Controlled Trials, and study registries (last search: July 2018) and screened reference lists of relevant systematic reviews and health technology assessments. Manufacturers and investigators were asked to provide unpublished data. Eligible studies investigated at least one patient-relevant outcome (e.g. wound closure). We assessed publication bias and, if feasible, performed meta-analyses, grading the results into different categories (hint, indication or proof of a greater benefit or harm).

**Results:**

We identified 48 eligible studies of generally low quality with evaluable data for 4315 patients and 30 eligible studies with missing data for at least 1386 patients. Due to potential publication bias (proportion of inaccessible data, 24%), we downgraded our conclusions. A meta-analysis of all wound healing data showed a significant effect in favour of NPWT (OR 1.56, 95% CI 1.15 to 2.13, *p* = 0.008). As further analyses of different definitions of wound closure did not contradict that analysis, we inferred an indication of a greater benefit of NPWT. A meta-analysis of hospital stay (in days) showed a significant difference in favour of NPWT (MD − 4.78, 95% CI − 7.79 to − 1.76, *p* = 0.005). As further analyses of different definitions of hospital stay/readmission did not contradict that analysis, we inferred an indication of a greater benefit of NPWT. There was neither proof (nor indication nor hint) of greater benefit or harm of NPWT for other patient-relevant outcomes such as mortality and adverse events.

**Conclusions:**

In summary, low-quality data indicate a greater benefit of NPWT versus SWT for wound closure in patients with wounds healing by secondary intention. The length of hospital stay is also shortened. The data show no advantages or disadvantages of NPWT for other patient-relevant outcomes. Publication bias is an important problem in studies on NPWT, underlining that all clinical studies need to be fully reported.

## Background

Chronic wounds affect about 1% of the population in Western industrialised countries, with much higher rates in inpatient settings, and pose a serious risk to patients’ health and quality of life [1–4]. Negative pressure wound therapy (NPWT), also called vacuum-assisted wound closure, was introduced into clinical practice in the early 1990s. With this technique, an open-cell foam dressing is placed into the wound cavity and a controlled subatmospheric pressure is applied to suck fluid from the wound, with the intention of improving wound healing [5]. In the past decades, the use of NPWT has increased considerably and it is currently applied across the world in both inpatient and outpatient settings for various surgical indications. Although multiple clinical benefits have been described, most clinical studies or evidence syntheses have failed to prove statistically significant or clinically relevant benefits versus standard wound therapy (SWT). For instance, in 2006, the German Institute for Quality and Efficiency in Health Care (Institut für Qualität und Wirtschaftlichkeit im Gesundheitswesen, IQWiG) conducted a health technology assessment (HTA) of NPWT studies [6] followed by a rapid report in 2007 [7] and found that “although there is some indication that NPWT may improve wound healing, the body of evidence available is insufficient to clearly prove an additional clinical benefit of NPWT. The large number of prematurely terminated and unpublished trials is the reason for concern” [8]. The IQWiG reports contained only a few small studies (all conducted in Western industrialised countries), all of poor methodological quality. In the meantime, considerably more evidence has accumulated on NPWT from randomised controlled trials (RCTs) conducted in multiple surgical indications and settings.

The aim of this systematic review of RCTs was therefore to assess the patient-relevant benefits and harms of NPWT versus SWT. Due to numerous and changing surgical indications and further developments in technology, our analysis considered all wounds healing by secondary intention.

## Methods

### Protocol and methodological approach

Our review formed part of a German-language HTA of the benefits and harms of NPWT in patients with wounds healing by secondary intention published by IQWiG in 2019. The full (German-language) protocol and report (Commission No. N17-01A) are available on the Institute’s website [9]. Both the preliminary protocol and the preliminary report underwent public commenting procedures. IQWiG’s responsibilities and methodological approach are described in its methods paper [10]. Only completed studies were used, so there was no need for ethical approval and patient consent. We adhered to the PRISMA statement [11] throughout this manuscript.

### Eligibility criteria

We included both published and previously unpublished RCTs comparing NPWT for wounds healing by secondary intention with any kind of SWT and investigating at least one predefined patient-relevant outcome. In this context, the term “patient-relevant” refers to “how a patient feels, functions or survives” [12]. The detailed eligibility criteria are presented in Table 1

**Table 1:** Eligibility criteria for studies included

Population	• Patients with wounds with intended secondary healing • Any healthcare setting
Study intervention	• Negative pressure wound therapy o No restrictions with regard to the use of commercial and/or custom-made devices o Type of further treatment, in particular indication for surgical wound closure, comparable to control intervention
Control intervention	• Standard wound therapy o Type of further treatment, in particular indication for surgical wound closure, comparable to study intervention
Patient-relevant outcomes	• Mortality • Wound closure • Adverse events • Amputation • Pain • Length of hospital stay and/or readmission to the hospital • Health-related quality of life • Physical function • Dependence on outside help or need for care
Study design	• Randomised controlled trials o Data of studies with fewer than 10 patients were excluded from the assessment. For transparency reasons those studies meeting the other eligibility criteria were included in the initial pool of relevant studies.
Publication type	• Availability of a full-text document (e.g. journal article or clinical study report, CSR) • No restrictions applied for the date of publication
Timing	• No restrictions
Language of publication	• Any language if English titles and abstracts were available and indicated potential relevance

### Search strategy and study selection

This systematic review is based on two previous HTA reports of IQWiG [6, 7]. We conducted an update search for the period not covered by these reports (from 2006 onwards). We searched the following bibliographic databases: MEDLINE, Embase, the Cochrane Central Register of Controlled Trials, the Cochrane Database of Systematic Reviews, and the Health Technology Assessment Database. The peer-reviewed search strategy included a combination of subject headings and free text with terms such as “negative pressure wound therapy” and “vacuum-assisted closure” (see Additional file 1 for the full search strategy). In addition, we searched ClinicalTrials.gov and the International Clinical Trials Registry Platform Search Portal. The last search was run on July 24, 2018. The reference lists of relevant systematic reviews and HTA reports published between 2013 and 2018 were scrutinised to identify further studies. In order to obtain the most complete data set possible, we also asked NPWT manufacturers to supply unpublished studies and additional unpublished data from published studies (see Additional file 1 for the full list of manufacturers).

As a prerequisite for the use of unpublished data, IQWiG asked the manufacturers to sign an agreement requiring (1) the submission of a list of all sponsored published and unpublished studies investigating NPWT and (2) the submission of CONSORT-compliant documents (in general the complete clinical study reports, CSRs) on all relevant studies selected by IQWiG. This procedure was required to avoid bias through the selective provision of data. Furthermore, we contacted the investigators responsible for investigator-initiated trials (IITs) to obtain the current study status or even data from potentially completed studies identified in study registries. In addition, persons and parties who had submitted comments on the preliminary version of the IQWiG report in the written public hearing were asked to provide any additional relevant studies.

Two reviewers independently screened titles and abstracts of the retrieved citations to identify potentially eligible primary and secondary publications. The full texts of these articles were obtained and independently evaluated by the same reviewers. All documents retrieved from non-bibliographical sources were also screened for eligibility or relevant information on studies. Disagreements were resolved by consensus.

Literature searching and study selection were done in parallel for two HTA reports, one on NPWT in patients with wounds healing by primary intention and one on NPWT in patients with wounds healing by secondary intention. The results of the HTA report on wounds healing by primary intention will be reported separately.

### Data extraction

The individual steps of the data extraction and risk-of-bias assessment procedures were always conducted by one person and checked by another; disagreements were resolved by consensus. Details of the studies were extracted using standardised tables.

We extracted information on:

1. Study characteristics, including the study design, length of follow-up, sample size, location, number of centres and period in which the study had been conducted.
2. Characteristics of the study participants, including inclusion and exclusion criteria, age, sex, wound characteristics at baseline, time since wound occurrence and dropout rate.
3. Characteristics of the test and control interventions, including treatment regimens and concomitant treatments.
4. Outcomes and type of outcome measures: outcomes as presented above; we did not limit the types of measures for a specific outcome, but rather analysed all measures used (e.g. wound healing (yes/no), time to wound healing).
5. Risk-of-bias items (see below).
  Information and data from publications were supplemented by publicly available results data from study registries and unpublished CSRs provided by manufacturers or IIT investigators.

### Assessment of risk of bias in individual studies

Using the IQWiG methods, we assessed the risk of bias (high or low) on the study and outcome level [10]. Because of the large number of studies, we conducted a stepwise assessment: if the generation of a randomisation sequence and/or the allocation concealment were inadequate, we assigned a high risk of bias to the study. If we did not, the following items were assessed at study level across outcomes: blinding of patients and treating staff, reporting of all relevant outcomes independent of results and other aspects, such as differences in the length of follow-up.

A high risk of bias on the study level generally led to a high risk of bias on the outcome level. Otherwise, the following outcome-specific items were assessed: blinding of outcome assessors, appropriate application of the intention-to-treat principle, reporting of individual outcomes independent of results and other aspects.

Using the IQWiG methods, we graded the results of the (meta-)analysis into different categories: proof, indication and hint (or neither proof, nor indication nor hint) of a greater benefit of the test intervention. In short, proof of a greater benefit of the test intervention is inferred if a meta-analysis of at least 2 studies with a low risk of bias shows a statistically significant effect favouring the test intervention. An indication of a greater benefit is inferred if one single study with a low risk of bias shows a statistically significant effect favouring the test intervention or a meta-analysis of studies with a high risk of bias shows a statistically significant effect favouring the test intervention. A hint of a greater benefit is inferred if a single study with a high risk of bias shows a statistically significant effect favouring the intervention. No proof (or indication or hint) of a greater benefit or harm is inferred if there are no statistically significant differences between the test and control interventions, if relevant heterogeneity exists or if no suitable data are available.

If studies with both a low and a high risk of bias are available for a specific outcome, the studies with a low risk of bias are primarily used to derive proof, an indication or a hint of a greater benefit of NPWT.

In addition to IQWiG methods, we also applied the GRADE approach (Grading of Recommendations Assessment, Development and Evaluation) in order to describe the certainty of the evidence in a widely used framework.

### Assessment of publication bias

Studies missing for analysis were defined as those that fulfilled the eligibility criteria listed above (except for the reporting of at least one patient-relevant outcome), had been completed at least 1 year before the last bibliographic and registry search and were not published. Studies that had been terminated prematurely or had an unclear study status (no update of study status in the 2 years before our literature search) were also counted as missing, as long as no contrary information was available (e.g. from author inquiries).

We assessed publication bias by comparing the estimated number of patients from missing studies with the number of all patients (from included and missing studies). If the proportion of missing data was < 10%, it was assumed that the impact of bias on the results introduced by the missing data was low and no action was taken. If the proportion of missing data was between 10 and 30%, it was assumed that the impact of bias on the results was high and all conclusions of proof, an indication or a hint of a greater benefit of NPWT were downgraded to an indication, a hint or no hint. If the proportion of missing data was > 30%, it was assumed that the impact of bias on the results was too high to be able to draw robust conclusions and no data analysis was performed.

### Data analysis

If results for different time points were available, the most recent one was used for the analysis, if not stated otherwise. The mean values and standard deviations of continuous variables were derived from the median, minimum and maximum values or the first and third quartile using the method by Wan 2014 [13] or from standard errors or confidence intervals (CI). If no information was available, missing standard deviations were derived from the median values of the standard deviations of all control interventions.

Odds ratios (OR) were calculated to compare dichotomously measured outcomes, mean differences (MD) or Hedges’ g were calculated to compare continuously measured outcomes. In most cases, Hedges’ g was used to adjust for different wound types, different scales applied for outcome measurements or heterogeneity in the original scale. For all effect estimates, 95% CI were reported.

If feasible and meaningful, data were pooled by means of meta-analyses. An overall effect was calculated using the Knapp and Hartung method with the Paule-Mandel heterogeneity estimator [14]. If only 2 studies were available, a fixed-effect model with inverse variance [15] was used to combine the study results. We used the beta-binomial model [16] to calculate an overall effect estimate to account for studies with no events in both treatment arms (double-zero studies).

If relevant heterogeneity [15] was present (*p* < 0.05), no overall effect estimate was calculated and, if possible, a 95% prediction interval (PI) [17] was calculated instead.

The results of the meta-analysis were presented in a forest plot. If studies with a low risk of bias showed a statistically significant effect, they were presented separately within the same plot.

A *p* value of < 0.05 was considered statistically significant.

We also planned subgroup analyses for age, sex, type of indication/wound, type of health care setting, and type of NPWT device.

## Results

### Literature search

**Fig. 1:**
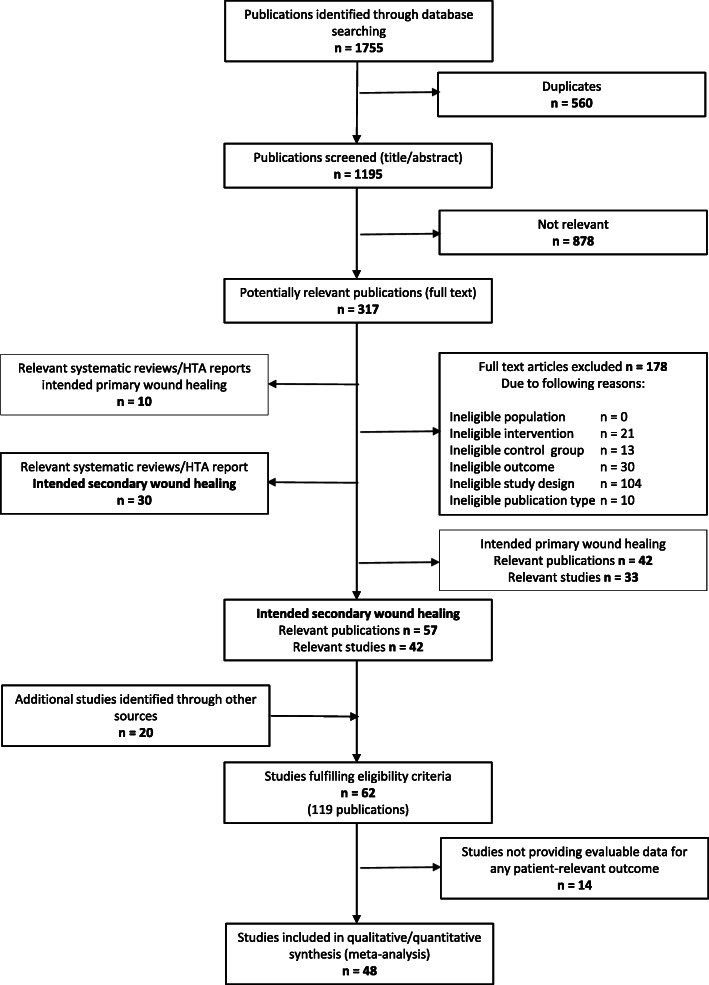
Flowchart of study selection (based on Moher et al. [11])

A total of 42 eligible studies were identified from 1195 references retrieved from bibliographic databases. Details of the study selection process from bibliographic databases are shown in Fig. 1. In addition, 20 potentially eligible studies were identified in further sources: 7 studies in the previous IQWIG reports, 5 in the reference lists of other relevant systematic reviews and one in a study registry. Two studies were identified in study registries and became eligible due to data provided by the investigator or manufacturer. Five additional and previously completely unpublished manufacturer-initiated studies finalised the initial study pool (details on data submission by the manufacturers are provided in the current IQWiG report [18]).

The 62 eligible studies (all RCTs) included 14 studies (ActiVac [19], Dwivedi 2016 [20, 21], Eginton 2003 [22], Ford 2002 [23], ISAW [24–26] [27], Joseph 2000 [28], Keskin 2008 [29], Riaz 2010 [30], Sajid 2015 [31], Sun 2007 [32], Vaidhya 2015 [33] and Wanner 2003 [34]) that failed to provide any evaluable data on patient-relevant outcomes. As these studies fulfilled the eligibility criteria and for transparency reasons, they were formally included in the initial study pool but excluded from the analysis. Ultimately, 48 eligible studies with evaluable data on 4315 patients were analysed.

### Potential publication bias

The literature search also identified 45 further studies without any published data on patient-relevant outcomes (of which 12 were planned or ongoing). These studies included 14 terminated, 9 completed studies and 10 studies with an unknown study status—30 out of these 33 studies should already have provided results, as the study had been completed at least 12 months before the search date of the present review. These 30 studies represented missing data of at least 1386 patients; for further details, see Table 2. Compared with the available evaluable data of 4315 patients, this results in a proportion of inaccessible data of 24% (1386/5701) of eligible patients. In consequence, we downgraded the certainty of our conclusions as described in the “Methods” section and refrained from doing subgroup analyses because the results would be hardly interpretable. Detailed documentation of all 45 studies without any published data on patient-relevant outcomes is given in Table 18 of the current IQWiG report [18].

**Table 2:** Unpublished studies considered for assessment of potential publication bias

Study	Sample size^**a**^	Documents available (e.g. study registry number, study protocol, CSR)	Recruitment status (estimated study completion date^**b**^)
ACTRN12614000056695	40	ACTRN12614000056695 [35]/--	Completed (December 2013)
Adams et al. (2005) [6]	1^c^	--/--	Completed (March 2005)^d^
ATEC	112	ISRCTN60292377 [36]/--	Completed (September 2016)
B2108R [6]	120	NCT00011531 [37]/--	Completed (December 2001)
CTRI/2018/01/011503	54	CTRI/2018/01/011503 [38]/--	Completed (April 2017)
foryou	48	ChiCTR-TRC-12002700 [39]/--	Completed (December 2015)^e^
VACOTOL-012	28	NCT02102685 [40]/--	Completed (September 2013)
VSD	119	ChiCTR-IOR-16008087 [41]/--	Completed (March 2016)^e^
045-1502-226 [6]	30	NCT00121537 [42]/--	Terminated (October 2015)^e^
2008/2023-31	30^f^	NCT01191567 [43]/--	Terminated^f^ (July 2012)
ANSM	36	NCT02509533 [44]/--	Terminated (July 2015)
Greer et al. (1999) [6]	16^g^	--/study protocol^h^ [45], raw data^h, i^ [46]	Terminated (November 1999)^g^
HTA012-0801-01	184	NCT00691821 [47]/--	Terminated (July 2011)^e^
STOMAVAC	14^g^	ISRCTN37399763 [48]/--	Terminated (December 2014)
U1111-1132-0768	30	ACTRN12612000702819 [49]/--	Terminated^f^ (n.s.)^J^
U1111-1133-5694	0^f^	ACTRN12612000885897 [50]/--	Terminated^f^ (n.s.)
U1111-1162-0654	16^f^	ACTRN12614001068651 [51]/--	Terminated^f^ (n.s.)^J^
VAC 2001-00 [6]	46	-- /study protocol^h^ [52], CSR^h,k^ [53]	Terminated^g^ (n.s.)^J^
VAC 2006-19	19^g^	NCT00837096 [54]/study protocol^h^ [55]	Terminated (October 2013)^e^
VAC TRIAL	9	ACTRN12606000384550 [56]/study protocol^h^ [57]	Terminated (September 2005)
2015046	80	NCT02374528 [58]/--	Unknown (April 2016)
382094-2	30	NCT01857128 [59]/--	Unknown ( December 2014)
ACTRN12609000149268	60	ACTRN12609000149268 [60]/--	Unknown^l^ (n.s.)^J^
ACTRN12609000995279	100	ACTRN12609000995279 [61]/--	Unknown^l^ (n.s.)^J^
CTRI/2014/02/004390	40	CTRI/2014/02/004390 [62]/--	Unknown^l^ (n.s.)^J^
Foo et al. (2004) [6]	--^m^	--/--	Unknown^d^ (n.s.)
Gupta et al. (2001) [6]	1^c^	--/--	Unknown^d^ (n.s.)
ITIQ002A	90	NCT01734109 [63]/--	Unknown (March 2014)
McCarthy M 2005	1^c^	--/--	Unknown (n.s.)^J^
NPWTvsGPA	32	NCT02314468 [64]/--	Unknown (October 2016)

*CSR* clinical study report, *n.s.* not specified

^a^Number of patients counted as missing; according to study registry information, if not stated otherwise

^b^According to study registry information, if not stated otherwise

^c^Not known; *N* = 1 used as a placeholder

^d^According to status of previous HTA report N04-03; no further information available

^e^Date of last study registry update; study may have been completed/terminated for a longer period of time

^f^According to author’s reply

^g^According to a manufacturer’s reply

^h^Not publically available

^i^Raw data provided by a manufacturer; refer to less than 70% of included patients; no data for planned patient-relevant outcomes included

^j^According to the available information study, study should have been completed/terminated for more than 12 months

^k^It is not possible to certainly assign the CSR provided by the manufacturer to the study under investigation. Furthermore, several pages had been deleted

^l^Classification as “unknown” as the status had not been updated within the 2 years before our literature search

^m^According to the previous HTA report N04-03 [6], change in wound surface should have been investigated. This outcome does not represent a patient-relevant outcome. The study was not further taken into account

### Characteristics of included studies

Table 3 presents the main characteristics of the 48 studies reporting evaluable results on patient-relevant outcomes. These studies included between 12 and 460 patients and were conducted worldwide between 1998 and 2016. The majority were 2-arm studies (*n* = 46); one study was 3-armed (Novinščak 2010 [112]) and one was 4-armed (TOPSKIN [122]). The study design was mostly monocentric (*n* = 35). The majority of studies were performed in an inpatient setting (*n* = 38). In 47 studies, patients were randomised and in one study (Moisidis 2004 [107]) wound halves were randomised. In 45 studies, one wound per patient and in 2 studies (Kakagia 2014 [100] and VAC 2001-06 [132]) at least one wound per patient was analysed.

**Table 3:** Characteristics of included studies

Study	Study design	***N*** participants randomised	Duration of active treatment (intervention group)	Study duration (including length of follow-up)	Setting	Location and study period	Relevant outcomes	Indication/wound type
Acosta et al. (2013) [65–67]	RCT Blinding not specified Single centre	20	Not specified	Until complete epithelization of the skin	Inpatient with outpatient continuatiOn	Sweden February 2007–April 2012	Mortality Wound closure Adverse events Amputation Hospital stay and readmission	Acute open wounds Deep peri-vascular groyne infections (Szilagyi grade III)
Arti et al. (2016) [68, 69]	RCT Blinding not specified Single centre	90	Generally 10–14 days	1 month	Inpatient	Iran February 2013–March 2015	Wound closure Adverse events	Acute open wounds Open fracture wound type IIIb based on Gustilo-Anderson classification
Ashby et al. (2012) [70, 71]	RCT Outcome-assessor blinded Single centre	12	According to the requirements of the nursing staff	6 months	Inpatient, nursing home and patient’s home	UK September 2008–August 2009	Mortality Wound closure Adverse events Pain	Chronic open wounds Grade III/V pressure ulcers according to the European Pressure Ulcer Advisory Panel Grading System
Banasiewicz et al. (2013) [72]	RCT Blinding not Specified Single centre	19	Not specified	Until the wounds healed to restore normal activity	Outpatient	Poland 2012	Pain Physical function	Acute open wounds Pilonidal sinus (primary/recurrent)
Bee et al. (2008) [73]	RCT Blinding not Specified Single centre	51	maximum 9 days	Not specified	Inpatient	USA April 2003–July 2007	Mortality Adverse events	Acute open wounds Temporary abdominal closure after damage control laparotomy, massive visceral oedema and planned reexploration
Biter et al. (2014) [74, 75]	RCT Unblinded Single centre	49	14 days	6 months after wound closure	Outpatient	Netherlands October 2009–May 2012	Wound closure Adverse events Pain Physical function	Acute open wounds Symptomatic pilonidal sinus with or without a previous abscess of the sinus
Braakenburg et al. (2006) [76]	RCT Blinding not Specified Single centre	64	Not specified	Until 80 days	Inpatient	Netherlands March 2002–May 2004	Mortality Wound closure Adverse events Amputation	Acute, subacute and chronic wounds Any type of wound
*CE/044/PIC* [77–82]	RCT Unblinded Multicentre (20 centres)	62	Until wound healing (maximum 12 weeks)	12 weeks	Inpatient, at home, medical practice and/or others	Canada and UKMarch 2012–October 2014	wound closure Adverse events pain Hospital stay and readmission	Subacute or chronic wounds (diabetic foot ulcer, pressure ulcer, venous leg ulcer or other chronic) suitable for treatment with NPWT
Chiang et al. (2017) [83]	RCT Unblinded Single centre	36	Not specified	12 months	Inpatient	New ZealandMarch 2010–June 2011	Adverse events	Acute open wounds Patients with high risk vascular foot wounds
Correa et al. (2016) [84, 85]	RCT Unblinded Single centre	75	Not specified	Until discharge from hospital	Inpatient	Columbia June 2011–April 2013	Mortality	Acute open wounds Traumatic open abdomen and open abdomen of a medical cause
Dalla Paola et al. (2010) S-II [86]	RCT Blinding not specified Single centre	130	Until wound healing or surgical wound closure	6 months	Inpatient	Italy July 2007–July 2008	Wound closure Adverse events amputation	Chronic open wounds Diabetic foot wounds
De Laat et al. (2011) [87, 88]	RCT Unblinded Single centre	24	Not specified	Maximum 6 weeks	Inpatient	Netherlands March 2003–March 2005	Adverse events	Chronic open wounds Difficult-to-heal surgical wounds or paraplegic and tetraplegic patients with pressure ulcers grade IV according to the European Pressure Ulcer Advisory Panel grading system
*DiaFu* [89–95]	RCT outcome-assessor blinded Multicentre (40 centres^b^)	368	Until wound healing or surgical wound closure (maximum 16 weeks)	6 months	In- and outpatient	Germany December 2011–February 2015	Mortality Wound closure Adverse events Amputation Pain	Chronic open wounds Diabetic foot lesions of stadium 2 to 4 according to the Wagner classification
Gupta et al. (2013) [96]	RCT blinding not Specified single centre	30	Not specified	Not specified	Inpatient	India Study period not specified	Wound closure Adverse events Hospital stay and readmission	Acute open wounds Open musculoskeletal injuries in extremities that required coverage procedures
Hu et al. (2009) [97]	RCT blinding not specified Single centre	67	Until complete wound healing	Until complete wound healing	Inpatient	China September 2005–November 2008	Wound closure Adverse events Amputation	Chronic open wounds Complex or refractory type lesions
Huang et al. (2006) [98]	RCT blinding not specified Single centre	24	Until wound closure	until natural surgery wound closure	inpatient	Taiwan 2004	Mortality Amputation Hospital stay and readmission	Acute open wounds Upper or lower limb of acute necrotizing fasciitis
Jayakumar et al. (2013) [99]	RCT Blinding not specified Single centre	40	Not specified	Not specified	Inpatient	India study period not specified	Wound closure Adverse events Hospital stay and readmission	Acute open wounds Type IIIA and Type IIIB open fracture both bones of leg
Kakagia et al. (2014) [100]	RCT Blinding not specified Single centre	50 (82 wounds)	Not specified	Average 21 months (range 5–36 months)	Inpatient	Greece June 2006–May 2011	Wound closure Adverse events	Acute open wounds Leg fasciotomies due to fractures and/or soft tissue injuries
Karatepe et al. (2011) [101]	RCT Blinding not specified Single centre	67	Not specified	Mean 4 months (range 2–8 months)	Inpatient	Turkey May 2007–December 2008	Wound closure	Chronic open wounds Biabetic foot ulcers
Leclercq et al. (2016) [102]	RCT Unblinded Single centre	46	5 days	3 months	Inpatient	France October 2010–May 2014	Wound closure	Surgically covered wounds Autologous grafting on chronic leg ulcers
Liao et al. (2012) [103]	RCT Blinding not specified Single centre	60	7–10 days	Average 24 months (range 12–36 months)	Inpatient	China March 2005–June 2010	Adverse events Hospital stay and readmission	Acute open wounds Amputation wounds for limbs open fractures
Llanos et al. (2006) [104]	RCT Outcome-assessor blinded Single centre	60	4 days	7–23 days	Inpatient	Chile May 2003–October 2004	Wound closure Adverse events Hospital stay and readmission	Acute open wounds Acute traumatic injuries and skin loss which hindered primary closure
Mody et al. (2008) [105]	RCT Outcome-assessor blinded Single centre	55	Until discharge from hospital	Average 26 days ± 18 days (intervention group) Average 33 days ± 37 days (control group)	In- and outpatient	India Study period not specified	Adverse events Amputation Pain	Acute and chronic open wounds Acute or chronic extremity sacral or abdominal wound that could not be treated with primary closure
Mohsin et al. (2017) [106]	RCT Outcome-assessor blinded Single centre	100	4 days	Until discharge from hospital	Inpatient	India January 2013–December 2015	Adverse events	Surgically covered wounds
Moisidis et al. (2004) [107]	RCT Outcome assessor blinded Single centre	22 (44 half wounds)	5 days	2 weeks	Inpatient	Australia July 2001–July 2002	wound closure Adverse events	acute or chronic open wounds Split-thickness skin graft on acute, subacute or chronic wounds
Mouës et al. (2004) [108–110]	RCT Blinding not specified Single centre	54	Until surgery wound closure	Until 1 month	Inpatient	Netherlands July 1998–October 2002	Mortality Wound closure Adverse events	Acute or chronic open wounds Full-thickness wounds
Nain et al. (2011) [111]	RCT Blinding not specified Single centre	30	Until wound closure (maximum 56 days)	Maximum 8 weeks	Inpatient	India Study period not specified	Wound closure	Chronic open woundsDiabetic foot ulcers
Novinščak et al. (2010) [112]	RCT 3 trial arms Blinding not specified Single centre	27^c^	Not specified	2 months	Inpatient	Croatia Study period not specified	Wound closure	Chronic open wounds Complicated diabetic ulcer (Wagner 2–5)
Perez et al. (2010) [113]	RCT Unblinded Single centre	49	Not specified	Until 30 days after wound healing	Inpatient	Haiti January 2007–June 2007	Wound closureAdverse events	Acute and chronic open wounds Fasciitis of leg or forearm, Fournier gangrene, abdominal wound, cervical wound, inguinal hernia repair, trauma to extremities, venous leg ulcer
Rencüzoğulları et al. (2015) [114]	RCT Blinding not specified Single centre	40	Not specified	Not specified^d^	Inpatient	Turkey February 2007–September 2010	mortality Adverse events Hospital stay and readmission	Acute open wounds Open abdomen/decompressive laparotomy as part of the management of abdominal compartment syndrome
Saaiq et al. (2010) [115]	RCT Patients blinded Single centre	100	10 days	Until wound healing	Inpatient	Pakistan October 2007–December 2009	Mortality Wound closure Adverse events Hospital stay and readmission	Acute open wounds Acute traumatic wounds most frequently located on the lower limb, upper limb, trunk and scalp
Shen et al. (2013) [116]	RCT Blinding not specified Single centre	307	6 days	Not specified^e^	Inpatient	China August 2009–May 2012	Wound closure	Acute open wounds Superficial partial thickness scald in children, shallow second degree burns mainly being located on the thorax, abdomen and limbs
Sibin et al. (2017) [117]	RCT Blinding not specified Single centre	30	Not specified	Not specified	Inpatient	IndiaJanuary 2015–July 2015	Wound closure Adverse events Hospital stay and readmission	Scute open wounds Gustilo type IIIA or IIIB open tibia fractures
Sinha et al. (2013) [118]	RCT Outcome assessor blinded Single centre	30	Not specified	Not specified^f^	Inpatient	India 2011–2012	Adverse events	Acute open wounds Open musculoskeletal injuries in extremities according to Gustilo Anderson classification grade II, IIIA, IIIB and IIIC
SWHSI [119–121]	RCT Outcome assessor blinded Multicentre (3 centres)	40	Not specified	3 months	In- and Outpatient	UK November 2015–September 2016	Wound closure Adverse events Amputation Pain Hospital stay and readmission Health-related quality of life	Acute open wounds Surgical wounds on the foot, abdomen, leg, breast, groyne, buttocks or perianal area
TOPSKIN [122–124]	RCT 4 trial Arms Unblinded Multicentre (3 centres)	86	Not specified	12 months	Inpatient	Netherlands October 2007–February 2010	Adverse events Pain Hospital stay and readmission	Acute open wounds Deep dermal or full-thickness burns of arm, leg or trunk requiring skin transplantation
*VAC* (*2001–01)* [125–127]	RCT Outcome-assessor blinded Multicentre (25 centres)	263^g^	Until surgery wound closure or wound healing with secondary intention (maximum 84 days)	Maximum 12 months	In- and outpatient	Canada and USA August 2001–October 2006	Mortality Adverse events	Chronic open wounds Stage III and IV pressure ulcers according to the National Pressure Advisory Panel (NPUAP) staging system located on the trunk or trochanter region
*VAC (2001–02)* [126, 128, 129]	RCT Outcome-assessor blinded Multicentre (29 centres)	208	Until wound healing with secondary intention (maximum 112 days)	Maximum 12 months	Inpatient^h^	USA January 2002–July 2005	Adverse events	Chronic open wounds Venous stasis ulcers
*VAC* (*2001–03)* [130, 131]	RCT Outcome-assessor blinded Multicentre	12	Not specified	90 days	Not specified	USA October 2001–July 2004	Wound closure Adverse events	Shronic open wounds Split thickness skin graft closure of venous stasis ulcers
VAC (2001–06) [132–134]	RCT Unblinded Single centre	58 (62 wounds)	Until surgery wound closure	Average 28 months (range 14–67 months)	Inpatient	USA June 2001–August 2006	Wound closure Adverse events Amputation Hospital stay and readmission	Acute open wounds Severe open fractures including type II fractures, type IIIA fractures that were either heavily contaminated or had a remarkably severe soft tissue injury, and all type IIIB or IIIC fractures according to the classification of Gustilo and Anderson
VAC (2001–07) [135–141]	RCT Outcome assessor blinded Multicentre (19 centres)	164^i^	Until wound closure (maximum 112 days)	Maximum 13 months^j^	Inpatient^h^	USA August 2002–November 2005	Mortality Wound closure Adverse events Hospital stay and readmission	Chronic open wounds Diabetic foot amputation wound up to the transmetatarsal region of the foot
VAC (2001–08) [142–149]	RCT Unblinded Multicentre (29 centres)	335	Until wound closure (maximum 112 days)	Maximum 12 months	Inpatient^h^	Canada and USA August 2002–August 2005	Mortality Wound closure Adverse events	Chronic open wounds Diabetic foot ulcer equivalent to Stage 2 or greater as defined by Wagner’s Scale
*VAC (2002–09)* [126, 150, 151]	RCT Outcome assessor blinded Multicentre (14 centres)	54	Until surgery wound closure or wound healing with secondary intention (maximum 84 days)	Maximum 6 months	Inpatient^h^	Canada and USA October 2002–July 2005	Mortality Wound closure Adverse events	Acute open wounds Open chest wounds
*VAC (2002–10)* [126, 152, 153]	RCT Outcome assessor blinded Multicentre (19 centres)	134	Until surgery wound closure or wound healing with secondary intention (maximum 84 days)	Maximum 6 months	Inpatient^h^	Canada, Mexico and USA June 2002–October 2004	Mortality Wound closure Adverse events	Acute open wounds Open abdominal wounds
Virani et al. (2016) [154]	RCT Blinding not specified Single centre	93	Until sufficient granulation tissue is present or approximation of the wound margins	Average 23 weeks ±6 weeks	Inpatient	India Study period not specified	Wound closure Adverse events	Acute open wounds Open diaphyseal tibial fractures, the majority of which were Gustilo Anderson Grade II and Grade IIIA fractures with heavy contamination and severe soft tissue and bony injury along with all Grade IIIB and Grade IIIC fractures
Vuerstaek et al. (2006) [155–157]	RCT Blinding not specified Multicentre (2 centres)	60	Maximum 4 days	12 months	Inpatient	Netherlands May 2001–May 2003	Mortality Wound closure Adverse events Pain Hospital stay and readmission	Chronic open wounds Chronic venous, combined venous and arterial, or microangiopathic (arteriolosclerotic) leg ulcers of > 6 months’ duration
WOLLF [158–161]	RCT Outcome assessor blinded Multicentre (24 centres)	460^k^	Until wound closure or surgical covering	12 months	Inpatient	UK 07/2012–2012/2015	Mortality Wound closure Adverse events Amputation Pain Health-related quality of life Physical function	Acute open wounds Severe open fracture of the lower limb. Wounds were graded as a Gustilo and Anderson II or III
Xu et al. (2015) [162]	RCT Blinding not specified Single centre	40	3–5 days	Not specified^l^	Inpatient	China 09/2013–2009/2014	Mortality Wound closure Adverse events Hospital stay and readmission	Acute open wounds Necrotizing fasciitis in the inguinal region or genital area

*Study title in italics :* study unpublished

*RCT* randomised controlled trial

^a^Data from www.ClinicalTrials.gov, 5 to 20 study centres are listed in the study protocol

^b^Number of study centres where patients were enrolled

^c^IQWiG’s own calculation

^d^The authors’ presentation indicates that the patients were observed until they were discharged from hospital. Accordingly, the intervention group was observed for an average of 28.5 days ±21.3 days and the control group for an average of 27.4 days ± 25.3 days

^e^The authors’ presentation indicates that the patients were observed until they were discharged from hospital. However, no further details can be found. Only the data on time to wound healing with an average of 9.2 days ± 0.6 days in the intervention group and an average of 10.1 days ± 1.6 days in the control group allow an approximate estimation of the study duration

^f^The authors’ presentation indicates that the patients were observed for 8 days

^g^Seven patients received no intervention

^h^The information provided indicates that at least outpatient aftercare was provided as part of the study. There are no explicit statements on the outpatient use of NPWT

^i^Two patients received no intervention

^j^For patients with wound healing. Patients without wound healing were not monitored after the maximum treatment duration of 112 days

^k^Originally, 625 patients were randomised, but due to the severity of the disease, only 460 patients were included in the study

^l^The authors' presentation indicates that the patients were observed until they were discharged from hospital. Accordingly, the intervention group was observed for an average of 21 days ±1.9 days and the control group for an average of 32 days ± 2.8 days

The 48 studies included covered a wide range of different wounds of various causes: amputation wounds (*n* = 1, Liao 2012 [103]), pressure ulcers (*n* = 2, Ashby 2012 [70], VAC 2001-01 [125]), diabetic foot wounds (*n* = 6, Dalla Paola 2010 S-II [86], DiaFu [89], Karatepe 2011 [101], Nain 2011 [111], VAC 2001-07 [135], VAC 2001-08 [142]), diabetic ulcer wounds (*n* = 1, Novinščak 2010), foot wounds (*n* = 1, Chiang 2017 [83]), fasciotomy wounds due to compartment syndrome (*n* = 1, Kakagia 2014), necrotizing fasciitis wounds (*n* = 2, Huang 2006 [98], Xu 2015 [162]), open fractures (*n* = 7, Arti 2016 [68], Gupta 2013 [96], Jayakumar 2013 [99], Sibin 2017 [117], VAC 2001-06, Virani 2016 [154], WOLLF [158]), open abdominal wounds (*n* = 4, Bee 2008 [73], Correa 2016 [84], Rencüzoğulları 2015 [114], VAC 2002-10 [152]), pilonidal sinus wounds (*n* = 2, Banasiewicz 2013 [72], Biter 2014 [74]), open thorax wounds (*n* = 1, VAC 2002-09 [150]), traumatic wounds of various causes (*n* = 3, Llanos 2006 [104], Saaiq 2010 [115], Sinha 2013 [118]), leg ulcer wounds (*n* = 4, Leclercq 2016 [102], VAC 2001-02 [128], VAC 2001-03 [130], Vuerstaek 2006 [155]), burns (*n* = 2, of which one was in infants, TOPSKIN, Shen 2013 [116]), groyne wounds caused by infection (*n* = 1, Acosta 2013 [65]), and various other wounds due to diseases and/or traumatic or iatrogenic causes (*n* = 10, Braakenburg 2006 [76], CE/044/PIC [77], De Laat 2011 [87], Hu 2009 [97], Mody 2008 [105], Moisidis 2004, Mouës 2004 [108], Mohsin 2017 [106], Perez 2010 [113], SWHSI [119]). Comparators were mostly described as standard wound care or standard dressings. If specified, dressings were described as sterilised gauze or moist gauze. Very few sudies provided more detailed information such as alginate, hydrofiber, silver-dressing or polyurethanes.

### Risk of bias in individual studies

The risk of bias at the study level was low in 7 (Ashby 2012, DiaFu, Llanos 2006, SWHSI, VAC 2001-07, Vuerstaek 2006 and WOLLF) out of 48 studies. As shown in Table 4, 41 studies were rated as having a high risk of bias at the study level due to an inadequate description of the randomisation procedure (*n* = 20) and/or the allocation concealment (*n* = 37), or selective reporting of outcomes (*n* = 4; TOPSKIN, VAC 2001-01, VAC 2001-02 and VAC 2001-08). Of the 7 studies rated as having a low risk of bias at the study level, only one also showed a low risk of bias for all outcomes (Llanos 2006). The studies by Ashby 2012 and SWHSI showed a low risk of bias, but not for all of the reported outcomes: pain, wound bleeding and infection (Ashby 2012) as well as adverse events, pain, duration of hospital stay and health-related quality of life (SWHSI) were rated as having a high risk of bias. In all other studies, the risk of bias at the outcome level was high. The detailed risk-of-bias assessments for all outcomes in all studies included are presented in Additional file 2.

**Table 4:** Risk of bias of included studies

Study	Randomisation appropriate	Allocation concealment appropriate	Blinding		Selective reporting improbable	Absence of other factors potentially causing bias	Risk of bias: study level ^**a**^	Risk of bias: outcome level^**b**^
			Patients	Treating staff				
Acosta et al. (2013) [65–67]	Yes	Unclear	n. a.	n. a.	n. a.	n. a.	High	High
Arti et al. (2016) [68, 69]	Unclear	Unclear	n. a.	n. a.	n. a.	n. a.	High	High
Ashby et al. (2012) [70, 71]	Yes	Yes	no	no	unclear	yes	Low	Low/high^c^
Banasiewicz et al. (2013) [72]	Unclear	Unclear	n. a.	n. a.	n. a.	n. a.	High	High
Bee et al. (2008) [73]	Unclear	Unclear	n. a.	n. a.	n. a.	n. a.	High	High
Biter et al. (2014) [74, 75]	Yes	Unclear	n. a.	n. a.	n. a.	n. a.	High	High
Braakenburg et al. (2006) [76]	Yes	Unclear	n. a.	n. a.	n. a.	n. a.	High	High
CE/044/PIC [77–82]	Unclear	Unclear	n. a.	n. a.	n. a.	n. a.	High	High
Chiang et al. (2017) [83]	Yes	Unclear	n. a.	n. a.	n. a.	n. a.	High	High
Correa et al. (2016) [84, 85]	Yes	Unclear	n. a.	n. a.	n. a.	n. a.	High	High
Dalla Paola et al. (2010) S-II [86]	Yes	Unclear	n. a.	n. a.	n. a.	n. a.	High	High
De Laat et al. (2011) [87, 88]	Unclear	Unclear	n. a.	n. a.	n. a.	n. a.	High	High
DiaFu [89–95]	Yes	Yes	no	no	yes	yes	Low	High
Gupta et al. (2013) [96]	Unclear	Unclear	n. a.	n. a.	n. a.	n. a.	High	High
Hu et al. (2009) [97]	Yes	Unclear	n. a.	n. a.	n. a.	n. a.	High	High
Huang et al. (2006) [98]	Unclear	Unclear	n. a.	n. a.	n. a.	n. a.	High	High
Jayakumar et al. (2013) [99]	Unclear	Unclear	n. a.	n. a.	n. a.	n. a.	High	High
Kakagia et al. (2014) [100]	Yes	Unclear	n. a.	n. a.	n. a.	n. a.	High	High
Karatepe et al. (2011) [101]	Yes	Unclear	n. a.	n. a.	n. a.	n. a.	High	High
Leclercq et al. (2016) [102]	Unclear	Unclear	n. a.	n. a.	n. a.	n. a.	High	High
Liao et al. (2012) [103]	Yes	Unclear	n. a.	n. a.	n. a.	n. a.	High	High
Llanos et al. (2006) [104]	Yes	Yes	no	no	unclear	yes	Low	Low
Mody et al. (2008) [105]	Yes	Unclear	n. a.	n. a.	n. a.	n. a.	High	High
Mohsin et al. (2017) [106]	Yes	Unclear	n. a.	n. a.	n. a.	n. a.	High	High
Moisidis et al. (2004) [107]	Unclear	Unclear	n. a.	n. a.	n. a.	n. a.	High	High
Mouës et al. (2004) [108–110]	Unclear	Unclear	n. a.	n. a.	n. a.	n. a.	High	High
Nain et al. (2011) [111]	Unclear	Unclear	n. a.	n. a.	n. a.	n. a.	High	High
Novinščak et al. (2010) [112]	Unclear	Unclear	n. a.	n. a.	n. a.	n. a.	High	High
Perez et al. (2010) [113]	Unclear	Unclear	n. a.	n. a.	n. a.	n. a.	High	High
Rencüzoğulları et al. (2015) [114]	Unclear	Unclear	n. a.	n. a.	n. a.	n. a.	High	High
Saaiq et al. (2010) [115]	Yes	Unclear	n. a.	n. a.	n. a.	n. a.	High	High
Shen et al. (2013) [116]	Yes	Unclear	n. a.	n. a.	n. a.	n. a.	High	High
Sibin et al. (2017) [117]	Unclear	Unclear	n. a.	n. a.	n. a.	n. a.	High	High
Sinha et al. (2013) [118]	Unclear	Unclear	n. a.	n. a.	n. a.	n. a.	High	High
SWHSI [119–121]	Yes	Yes	no	no	yes	yes	Low	Low/high^d^
TOPSKIN [122–124]	Unclear	Yes	no	no	no	yes	High	High
VAC (2001–01) [125–127]	Yes	Yes	no	no	no	no^e^	High	High
VAC (2001–02) [126, 128, 129]	Yes	Yes	no	no	no	no^f^	High	High
VAC (2001–03) [130, 131]	Unclear	Unclear	n. a.	n. a.	n. a.	n. a.	High	High
VAC (2001–06) [132–134]	Yes	Unclear	n. a.	n. a.	n. a.	n. a.	High	High
VAC (2001–07) [135–141]	Yes	Yes	No	No	Yes	Yes	Low	High
VAC (2001–08) [142–149]	Yes	Yes	No	No	No	Yes	High	High
VAC (2002–09) [126, 150, 151]	Yes	Unclear	n. a.	n. a.	n. a.	n. a.	High	High
VAC (2002–10) [126, 152, 153]	Yes	Unclear	n. a.	n. a.	n. a.	n. a.	High	High
Virani et al. (2016) [154]	Yes	Unclear	n. a.	n. a.	n. a.	n. a.	High	High
Vuerstaek et al. (2006) [155–157]	Yes	Yes	No	No	Yes	Yes	Low	High
WOLLF [158–161]	Yes	Yes	No	No	Unclear	Yes	Low	High
Xu et al. (2015) [162]	Unclear	Unclear	n. a.	n. a.	n. a.	n. a.	High	High

*n. a.* not applied

^a^If the evaluation of the items “random sequence generation“ and “allocation concealment“ revealed a high risk of bias, no further evaluations of the remaining items were performed

^b^Details are given in Tables 1 to 8 in Additional file 2

^c^The outcomes pain and adverse events (wound bleeding and infection) showed a high risk of bias

^d^The outcomes adverse events, pain, duration of hospital stay and quality of life showed a high risk of bias

^e^In the control group and in the NPWT group, 27.8% and 7.7% of the patients respectively discontinued the study due to treatment failure. The time point of study discontinuation was documented as the final study visit date

^f^Only data from 146 out of 205 randomised patients available (71.2%)

### Effects of NPWT versus SWT

*Wound closure* was measured as wound healing (yes/no), time to wound healing (in days), wound healing and/or surgical wound closure (yes/no), time to wound healing and/or surgical wound closure (in days), time to wound healing after the intervention and surgical wound closure (< 6 weeks yes/no), and time to wound healing after the intervention and surgical wound closure (in days).

**Fig. 2:**
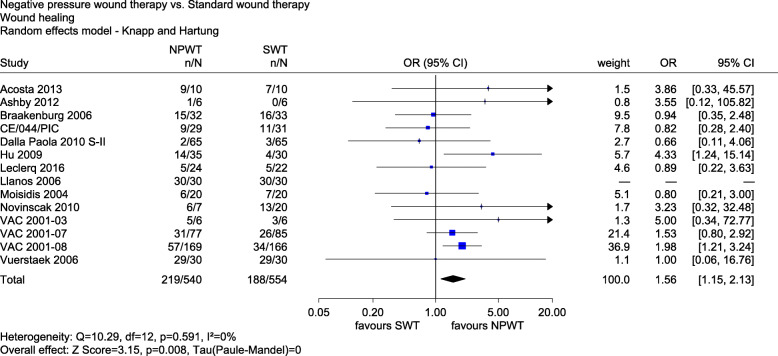
Forest plot of wound healing with overall effect estimation, NPWT vs. SWT. Abbreviations: CI confidence interval, *n* number of events, *N* number of patients, NPWT negative pressure wound therapy, OR odds ratio, SWT standard wound therapy

The results for *wound healing (yes/no)* were reported in 14 studies (Acosta 2013, Ashby 2012, Braakenburg 2006, CE/044/PIC, Dalla Paola 2010 S-II, Hu 2009, Leclercq 2016, Llanos 2006, Moisidis 2004, Novinščak 2010, VAC 2001-03, VAC 2001-07, VAC 2001-08 and Vuerstaek 2006). Two studies with a low risk of bias (Ashby 2012 and Llanos 2006) showed no statistically significant difference between the groups. The combined analysis of studies with a low and high risk of bias showed a statistically significant effect in favour of NPWT (OR 1.56, 95% CI 1.15 to 2.13, *p* = 0.008, see Fig. 2).

Only 7 studies defined wound healing as 100% re-epithelialization (Acosta 2013, CE/044/PIC, Dalla Paola 2010 S-II, Moisidis 2004, VAC 2001-07, VAC 2001-08 and Vuerstaek 2006), while in the other 7, a definition was missing. We therefore conducted a sensitivity analysis with the definition of wound healing as a stratification factor. Studies with a proper definition showed a statistically significant effect in favour of NPWT (OR 1.53, 95% CI 1.04 to 2.23, *p* = 0.034). Studies without such a definition showed no statistically significant effect (OR 1.72, 95% CI 0.73 to 4.04, *p* = 0.163). However, the interaction test showed no statistically significant difference between the two effects (*p* = 0.743). There was an indication of a greater effect of NPWT on wound healing.

**Fig. 3:**
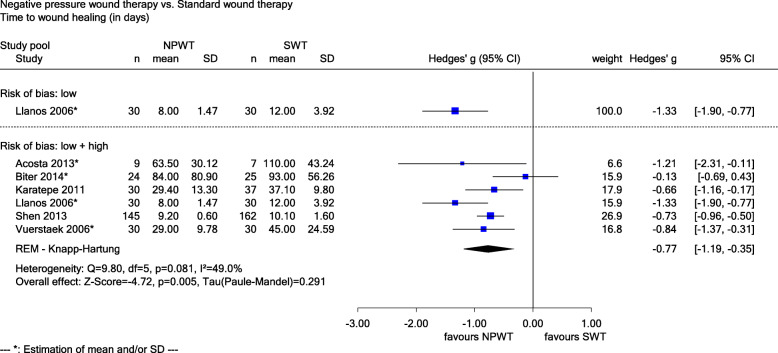
Forest plot of time to wound healing (in days) with overall effect estimation, NPWT vs. SWT. Abbreviations: CI confidence interval, *n* number of patients, NPWT negative pressure wound therapy, SD standard deviation, SWT standard wound therapy

Six studies reported data on *time to wound healing (in days)* (Acosta 2013, Biter 2014, Karatepe 2011, Llanos 2006, Shen 2013 and Vuerstaek 2006). The study with a low risk of bias (Llanos 2006) (Hedges’ g − 1.33, 95% CI − 1.90 to − 0.77, see Fig. 3), as well as studies with a low and high risk of bias (Hedges’ g − 0.77, 95% CI − 1.19 to − 0.35, *p* = 0.005, see Fig. 3) showed a statistically significant difference in favour of NPWT. The results were classified as clinically relevant, since the upper limits of these 95% CIs were below the irrelevance threshold of − 0.2. A sensitivity analysis stratified by the definition of wound healing showed a statistically significant result for the 4 studies (Acosta 2013, Biter 2014, Shen 2013 and Vuerstaek 2006) with a proper definition (100% re-epithelialization; Hedges’ g − 0.69, 95% CI − 0.88 to − 0.49, *p* < 0.001) and the 2 studies with a missing definition (Hedges’ g − 0.95, 95% CI − 1.33 to − 0.58, *p* < 0.001). However, the interaction test showed no statistically significant difference between the two effects (*p* = 0.210). There was proof of a greater effect of NPWT on time to wound healing.

**Fig. 4:**
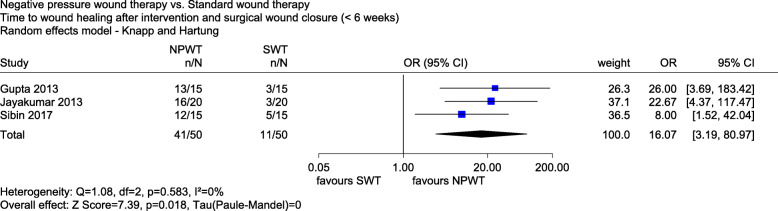
Forest plot of time to wound healing after intervention and surgical wound closure (< 6 weeks) with overall effect estimation, NPWT vs. SWT. Abbreviations: CI confidence interval, *n* number of events, *N* number of patients, NPWT negative pressure wound therapy, *OR* odds ratio, SWT standard wound therapy

The meta-analysis of *time to wound healing after the intervention and surgical wound closure (< 6 weeks yes/no)* included 3 studies with a high risk of bias (Gupta 2013, Jayakumar 2013 and Sibin 2017). There was a statistically significant effect in favour of NPWT after 6 weeks (OR 16.07, 95% CI 3.19 to 80.97, *p* = 0.018, see Fig. 4).

**Fig. 5:**
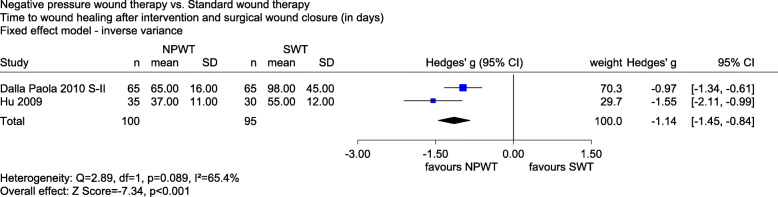
Forest plot of time to wound healing after the intervention and surgical wound closure (in days) with overall effect estimation, NPWT vs. SWT. Abbreviations: CI confidence interval, *n* number of patients, NPWT negative pressure wound therapy, SD standard deviation, SWT standard wound therapy

The meta-analysis of *time to wound healing after the intervention and surgical wound closure (in days)* included 2 studies with a high risk of bias (Dalla Paola 2010 S-II and Hu 2009). There was a statistically significant difference in favour of NPWT (Hedges’ g − 1.14, 95% CI − 1.45 to − 0.84, *p* < 0.001, see Fig. 5). The effect was clinically relevant, since the upper limit of the 95% CI was below the irrelevance threshold of − 0.2. Overall, there was an indication of a greater effect of NPWT on time to wound healing after the intervention and surgical closure.

Due to heterogeneity, no effect estimate was calculated for *wound healing and/or surgical wound closure (yes/no)* (21 studies; Acosta 2013, Arti 2016, Braakenburg 2006, Dalla Paola 2010 S-II, DiaFu, Gupta 2013, Hu 2009, Jayakumar 2013, Kakagia 2014, Mouës 2004, Nain 2011, Perez 2010, Saaiq 2010, Sibin 2017, SWHSI, VAC 2001-06, VAC 2001-07, VAC 2001-08, Virani 2016, WOLLF and Xu 2015) and *time to wound healing and/or surgical wound closure (in days)* (9 studies; Braakenburg 2006, Mouës 2004, Perez 2010, VAC 2001-07, VAC 2001-08, VAC 2002-09, VAC 2002-10, Virani 2016 and Vuerstaek 2006). In both analyses, the 95% PI included the zero effect (OR = 1 and Hedges’ g = 0); see Fig. 1 and 2 in Additional file 3. The heterogeneity for time to wound healing and/or surgical wound closure (in days) was mainly caused by Perez 2010. In a sensitivity analysis, the result of this study was shifted closer to zero, reducing heterogeneity. The mean value in the NPWT group was shifted towards the mean value in the SWT group until the test of heterogeneity was non-significant (*p* > 0.05). The resulting pooled effect was statistically significant (Hedges’ g − 0.39, 95 % CI − 0.66 to − 0.11, *p* = 0.013) but not clinically relevant, as the upper limit of the 95% CI was not below the irrelevance threshold of − 0.2. There was neither proof (nor indication nor hint) of a greater or smaller effect of NPWT on wound healing and/or surgical wound closure (yes/no) and time to wound healing and/or surgical wound closure (in days).

Overall, there was proof of a greater benefit of NPWT for wound closure. Due to the potential publication bias mentioned above, this conclusion was downgraded. There was thus an indication of a greater benefit of NPWT for wound closure.

*Adverse events* comprised additional measures required for direct wound closure (such as skin transplantation or sutures), re-interventions (such as regrafting or revision fixation), bleeding, infections, the overall rate of serious adverse events and study discontinuation due to adverse events.

Ten studies provided data on re-interventions (Chiang 2017, De Laat 2011, Liao 2012, Llanos 2006, Mohsin 2017, Moisidis 2004, Saaiq 2010, VAC 2001-07, VAC 2001-08 and WOLLF). One study with a low risk of bias (Llanos 2006) showed no statistically significant difference between groups. In the combined analysis of studies with a low and high risk of bias, NPWT significantly reduced the odds for re-interventions (OR 0.46, 95% CI 0.24 to 0.86, *p* = 0.021, see Fig. 3 in Additional file 3). One study (Perez 2010) measured the number of operations until wound closure and found a statistically significant difference in favour of SWT (MD 2.80, 95% CI 0.79 to 4.81, *p* = 0.008). Due to the small number of patients in this study, the result of the meta-analysis was not challenged. There was thus an indication of a greater effect of NPWT on re-intervention.

**Fig. 6:**
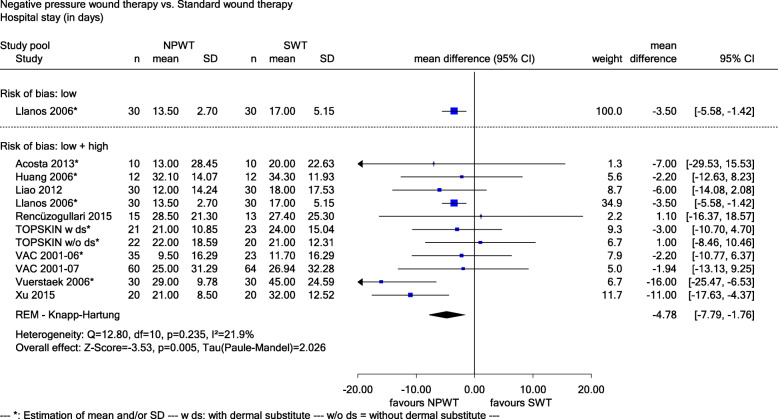
Forest plot of hospital stay (in days) with overall effect estimation, NPWT vs. SWT. Abbreviations: CI confidence interval, *n* number of patients, NPWT negative pressure wound therapy, SD standard deviation, SWT standard wound therapy

**Fig. 7:**
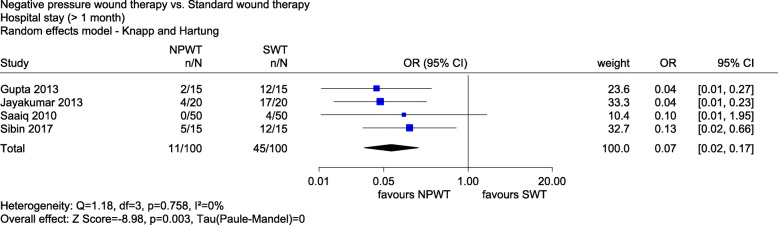
Forest plot of hospital stay (> 1 month) with overall effect estimation, NPWT vs. SWT. Abbreviations: CI confidence interval, *n* number of events, *N* number of patients, NPWT negative pressure wound therapy, OR odds ratio, SWT standard wound therapy

There were no statistically significant differences between groups with regard to additional measures required for direct wound closure (23 studies, see Fig. 4 in Additional file 3) and the overall rate of serious adverse events (12 studies, see Fig. 5 in Additional file 3). No effect estimate was calculated for infection (20 studies, see Fig. 6 in Additional file 3) and study discontinuation due to adverse events (7 studies, see Fig. 7 in Additional file 3) because of heterogeneity. In these 2 analyses, the 95% PI included the zero effect (OR = 1). For bleeding (5 studies, see Fig. 8 in Additional file 3), no events occurred in 3 out of 6 studies. The other 3 studies showed no statistically significant differences. No overall effect was therefore calculated. There was neither proof (nor indication nor hint) of a greater or smaller effect of NPWT on any of these outcomes. As the overall rate of serious adverse events was the primary analysis for adverse events, there was neither proff (nor indication nor hint) of greater benefit or harm of NPWT for adverse events.

*Hospital stay and readmission* was measured as hospital stay (in days) in 10 studies (Acosta 2013, Huang 2006, Liao 2012, Llanos 2006, Rencüzoğulları 2015, TOPSKIN, VAC 2001-06, VAC 2001-07, Vuerstaek 2006 and Xu 2015), hospital stay (> 1 month yes/no) in 4 studies (Gupta 2013, Jayakumar 2013, Saaiq 2010 and Sibin 2017), intensive care unit stay (in days) in 2 studies (Rencüzoğulları 2015 and TOPSKIN) and readmission (yes/no) in 4 studies (CE/044/PIC, De Laat 2011, SWHSI and VAC 2001-07). For all outcomes and studies (except one [Llanos 2006]), the risk of bias was high.

For hospital stay (in days), the study with a low risk of bias (MD − 3.50, 95% CI − 5.58 to − 1.42, see Fig. 6), as well as studies with a high and low risk of bias (MD − 4.78, 95% CI − 7.79 to − 1.76, *p* = 0.005, see Fig. 6) showed a statistically significant difference in favour of NPWT. There was a proof of a greater effect of NPWT on hospital stay (in days).

Hospital stay (> 1 month yes/no) also showed a statistically significant effect in favour of NPWT (OR 0.07, 95% CI 0.02 to 0.17, *p* = 0.003, see Fig. 7). There was an indication of a greater effect of NPWT on hospital stay (> 1 month).

Despite homogeneity between the studies (*I*^2^ = 0%, *p* = 0.441), no effect estimate was presented for intensive care unit stay (in days) because of the large range of the 95% CI (containing the zero effect MD = 0). There was no statistically significant effect for readmission (yes/no) (OR 1.01, 95% CI 0.44 to 2.31, *p* = 0.973, see Fig. 9 in Additional file 3). There was neither proof (nor indication nor hint) of a greater or smaller effect of NPWT on the 2 latter outcomes.

Overall, there was proof of a greater benefit of NPWT for hospital stay and readmission. Due to the high risk of publication bias, the evidence for this outcome was downgraded. There was thus an indication of a greater benefit of NPWT for hospital stay and readmission.

Data on *mortality* were available in 18 studies (Acosta 2013, Ashby 2012, Bee 2018, Braakenburg 2006, Correa 2016, DiaFu, Huang 2006, Mouës 2004, Rencüzogullari 2015, Saaiq 2010, VAC 2001-01, VAC 2001-07, VAC 2001-08, VAC 2002-09, VAC 2002-10, Vuerstaek 2006, WOLLF and Xu 2015). There was no statistically significant effect of NPWT compared to SWT (OR 1.20, 95 % CI 0.84 to 1.70, *p* = 0.290, see Fig. 10 in Additional file 3).

Data on *amputation* were available in 10 studies (Acosta 2013, Braakenburg 2006, Dalla Paola 2010 S-II, DiaFu, Hu 2009, Huang 2006, Mody 2008, SWHSI, VAC 2001-06 and WOLLF). There was no statistically significant effect of NPWT compared to SWT (OR 0.89, 95% CI 0.55 to 1.43, *p* = 0.588, see Fig. 11 in Additional file 3).

*Pain* was measured continuously in 6 studies (visual analogue scale [Banasiewicz 2013, Biter 2014, SWHSI and Vuerstaek 2006], visual analogue thermometer [TOPSKIN], numeric rating scale [DiaFu]), dichotomously (pain yes/no) in 3 studies (Ashby 2012, Mody 2008 and WOLLF), and as pain on application and removal of dressing (continuously [SWHSI], dichotomously [CE/044/PIC]). None of these outcomes showed a statistically significant difference between NPWT and SWT (see Figs. 12 and 13 and eTable 1 and eTable 2 in Additional file 3).

Two studies reported data on *health-related quality of life* using the Physical Composite Scale (PCS) and the Mental Health Composite Scale (MCS) derived from the SF-12 after 3 months (SWHSI) and 12 months (WOLLF), respectively. There was no statistically significant difference between NPWT and SWT for the MCS; heterogeneity was shown for the PCS (see Figs. 14 and 15 in Additional file 3).

Three studies provided data on *physical function* measured as time to resume work or school (Biter 2004), time to restoration of normal activity (Banasiewicz 2013) and the Disability Rating Index (DRI; [WOLLF]). A meta-analysis of the first 2 outcomes showed heterogeneity, while the DRI showed no statistically significant difference between NPWT and SWT (see Fig. 16 and eTable 3 in Additional file 3).

No study reported data on *dependence on outside help or need for care*. There was neither proof (nor indication nor hint) of a greater benefit or harm of NPWT for mortality, amputation, health-related quality of life, physical function and dependence on outside help or need for care.

Using the beta-binomial model to account for double-zero studies did not alter the results of the meta-analyses presented.

A summary of the results is presented in Table 5. An overview of the key findings according to GRADE methods can be found in Additional file 4.

**Table 5:** Summary of results

Outcome	Results	Grading of results
**Mortality**	OR 1.20, 95% CI 0.84 to 1.70, *p* = 0.290	⇔
**Wound closure**		⇑^a^
Wound healing (yes/no)	OR 1.56, 95% CI 1.15 to 2.13, *p* = 0.008	⇑
Time to wound healing (in days)	*Studies with low risk of bias:* Hedges’ g − 1.33, 95% CI − 1.90 to − 0.77 *Studies with low and high risk of bias:* Hedges’ g − 0.77, 95% CI − 1.19 to − 0.35, *p* = 0.005	⇑⇑
Time to wound healing after intervention and surgical wound closure	*< 6 weeks yes/no:* OR 16.07, 95% CI 3.19 to 80.97, *p* = 0.018 *In days:* Hedges’ g − 1.14, 95% CI − 1.45 to − 0.84, *p* < 0.001	⇑
Wound healing and/or surgical wound closure (yes/no)	Heterogeneous effects (OR) 95% PI 0.37 to 4.97	⇔
Time to wound healing and/or surgical wound closure (in days)	Heterogeneous effects (Hedges’ g) 95% PI − 3.47 to 2.10	⇔
**Adverse events**		⇔^b^
Additional measures required for direct wound closure	OR 1.20, 95% CI 0.70 to 2.08, *p* = 0.476	⇔
Re-interventions	*Yes/no:* OR 0.46, 95% CI 0.24 to 0.86, *p* = 0.021 *Number of re-interventions:* MD 2.80, 95% CI 0.79 to 4.81, *p* = 0.008	⇑^c^
Bleeding	No effect estimate (OR) is given due to 3 out of 6 studies without events and 3 studies with no statistically significant effects.	⇔
Infection	Heterogeneous effects (OR) 95% PI 0.07 to 5.21	⇔
Overall rate of SAEs	OR 1.02, 95% CI 0.76 to 1.37, *p* = 0.860	⇔
Study discontinuation due to AEs	Heterogeneous effects (OR) 95% PI 0.36 to 13.57	⇔
**Amputation**	OR 0.89, 95% CI 0.55 to 1.43, *p* = 0.588	⇔
**Pain**		⇔
Continuous	Hedges’ g − 0.16, 95% CI − 0.53 to 0.21, *p* = 0.32	⇔
Dichotomous	No effect estimate (OR) is given due to the large range of the 95% CI containing the zero effect (OR = 1).	⇔
Pain on application and removal of dressing	*Continuous:* MD − 0.30, 95% CI − 19.75 to 19.15, *p* = 0.975 *Dichotomous:* No statistically significant effects (OR) for all given weeks	⇔
**Hospital stay and readmission**		⇑^a^
Hospital stay (in days)	*Study with low risk of bias:* MD − 3.50, 95% CI − 5.58 to − 1.42 *Studies with low and high risk of bias:* MD − 4.78, 95% CI − 7.79 to − 1.76, *p* = 0.005	⇑⇑
Hospital stay (> 1 month yes/no)	OR 0.07, 95% CI 0.02 to 0.17, *p* = 0.003	⇑
Intensive care unit stay (in days)	No effect estimate (MD) is given due to the large range of the 95% CI containing the zero effect (MD = 0).	⇔
Readmission (yes/no)	OR 1.01, 95% CI 0.44 to 2.31, *p* = 0.973	⇔
**Health-related quality of life**		⇔
SF-12 MCS	Hedges’ g 0.01, 95% CI − 0.20 to 0.22, *p* = 0.937	⇔
SF-12 PCS	Heterogeneous effects (Hedges’ g)	⇔
**Physical function**		⇔
Time to resume work or school/restoration of normal activity (in days)	Heterogeneous effects (Hedges’ g)	⇔
DRI	No statistically significant effects (MD) for all given months	⇔
**Dependence on outside help or need for care**	No data	⇔

*AE* adverse event, *CI* confidence interval, *DRI* Disability Rating Index, *MCS* mental health composite scale, *MD* mean difference, *NWPT* negative pressure wound therapy, *OR* odds ratio, *p p* value, *PCS* physical composite scale, *PI* prediction interval, *SAE* serious adverse event, *SF* short for

^a^The proof was downgraded due to high risk of publication bias

^b^The overall rate of SAEs was the primary analysis of adverse events

^c^The indication of a greater effect of NWPT measured as re-intervention (yes/no) was not challenged by the hint of a smaller effect of NWPT measured as number of re-interventions

⇑⇑ Proof of a greater effect/benefit

⇑ Indication of a greater effect/benefit

⇗ Hint of a greater effect/benefit

⇔ No proof (or indication or hint) of a greater or smaller effect/of a greater benefit or harm

## Discussion

### Summary of findings

This systematic review of NPWT versus SWT in patients with wounds healing by secondary intention showed some advantages of NPWT with regard to wound closure and hospital stay. No differences were shown for any other important outcomes such as infection or amputation (see Table 5).

### Comparison with previous research

It is difficult to compare the present results with previous systematic reviews on NPWT due to their more or less restricted focus, in contrast to the rather wide question of the present review, which is wounds healing by secondary intention. Using only publications from 2013 to 2018, we identified 30 systematic reviews on NPWT for various wounds healing by secondary intention (see Additional file 5). Only 13 analysed wound closure, the key outcome for this type of intervention. Of these, 10 came to a positive conclusion [163–172], while 3 did not [173–175]. More importantly, only 14 [163–166, 168, 169, 171, 173, 175–180] of the 30 previous systematic reviews at least mentioned the risk of publication bias and none implemented any consequences for their conclusions in the event of this type of bias.

### Data pooling

The question as to whether to pool clinical study data or not is fundamental in meta-analysis. In the assessment of NPWT, various medical aspects need to be considered before pooling the data into a common effect estimate. The type of wound investigated is the most obvious difference between the studies in our analysis. Further factors potentially affecting study results are the exact type of NPWT technique used (e.g. pressure applied), choice of control treatment, type of healthcare setting and study duration. However, against the background of the lack of a standardised nomenclature for wounds and the fact that if the underlying disease and the respective wound is prepared optimally, wounds are very similar, it seemed meaningful to pool the data with regard to the type of wound healing. Furthermore, innovation would become impossible if each new wound treatment had to be tested for each of the numerous different wound types. Since several meta-analyses showed no or only little heterogeneity, our data at least do not contradict this approach.

Published evidence tends to overestimate the benefits and underestimate the harms of medical interventions [181], and it is widely accepted that the results of all relevant studies must be fully available for an unbiased estimation of effects. The introduction of mandatory measures such as registration of studies and their results has increased data availability, but many studies (in the present case mostly IITs), especially on medical devices, still remain partly or fully unpublished. Further measures to ensure full disclosure are thus urgently required. As the present case shows that voluntary commitment does not work, further legal and regulatory action, in combination with sanctions, seems to be indispensable. For instance, IQWiG proposes that funders of clinical research and ethic committees exert stronger supervision over research projects by denying further funding if previous projects were not properly published [18].

### Strengths and limitations

Strengths of the present research include the systematic literature search and the inclusion of several unpublished studies, which were in particular obtained from several NPWT manufacturers (and sometimes provided only after public pressure). This methodological rigour was essential in order to limit potential publication bias. Nevertheless, the present review included an insufficient proportion of the relevant clinical study data to draw highly robust conclusions, which is why our conclusions are cautious. In addition, we did not assess outcome reporting bias. We cannot exclude that the proportion of missing data would have been even higher if this type of bias had also been considered. It should also be noted that overall, the quality of the studies considered was low and the sample sizes were small. If larger, independently funded, multicentre trials had been performed, evaluation of NPWT would have been possible in an earlier, easier and more reliable way.

### Implications for future research

The advantages of NPWT were modest, and the main conclusion with regard to wound closure was derived from only 14 of the 48 studies. Due to various definitions of wound closure and different time points of data collection (or even missing information on these items), no conclusions can be made with regard to the sustainability of wound closure. In addition, as stated, the size and quality of the studies were generally low. Therefore, it is certainly not unethical to conduct further (but high-quality) RCTs on this topic. We recommend that they systematically investigate and clearly define the key outcomes of wound healing, adverse events and health-related quality of life.

## Conclusion

In summary, low-quality data indicate a greater benefit of NPWT versus SWT for the outcome of wound closure in patients with wounds healing by secondary intention. The length of hospital stay is also shortened. However, the data show no advantages or disadvantages of NPWT versus SWT for mortality, adverse events, amputation, pain or health-related quality of life. Although data on serious adverse events were not systematically collected in most of the primary studies, NPWT appears to be safe. Publication bias is an important problem in NPWT research, underlining that all clinical studies need to be fully reported regardless of funding source, premature study termination or study results.

## Supplementary information

**Additional file 1.** Search strategies applied and manufacturers contacted (DOCX 27 kb)

**Additional file 2.** Detailed risk of bias for assessments of outcomes (DOCX 49 kb)

**Additional file 3.** Forest plots not presented in the manuscript (DOCX 62 kb)

**Additional file 4.** Overview of key findings according to GRADE (DOCX 18 kb)

**Additional file 5.** List of previous systematic reviews (DOCX 18 kb)

## Data Availability

All data used in this article are available in the full German-language report published on the IQWiG website [9].

## Abbreviations

CI: Confidence interval
CSR: Clinical study report
DRI: Disability Rating Index
HTA: Health technology assessment
IIT: Investigator-initiated trial
MCS: Mental Health Composite Scale
MD: Mean difference
NPWT: Negative pressure wound therapy
OR: Odds ratio
PI: Prediction interval
PCS: Physical Composite Scale
PRISMA: Preferred Reporting Items for Systematic Reviews and Meta-Analyses
RCT: Randomised controlled trial
SWT: Standard wound therapy
