# Supplementary material for: Negative pressure wound therapy in patients with wounds healing by secondary intention: a systematic review and meta-analysis of randomised controlled trials
Source: Syst Rev. 2020 Oct 10;9:238. doi: 10.1186/s13643-020-01476-6 (PMC7548038; doi:10.1186/s13643-020-01476-6)
Supplement: Supplementary file 1 — Additional file 1. Search strategies applied and manufacturers contacted (DOCX 27 kb) [file 13643_2020_1476_MOESM1_ESM.docx]

**Additional file 1: Search strategies applied and manufacturers contacted**

**Search strategies**

Medline via Ovid

- Ovid MEDLINE(R) 1946 to July Week 2 2018
- Ovid MEDLINE(R) In-Process & Other Non-Indexed Citations July 20, 2018
- Ovid MEDLINE(R) Daily Update July 20, 2018

Ovid MEDLINE(R) Epub Ahead of Print July 20, 2018

Search filters:

- Systematic Reviews: Wong [1] – High specificity strategy

RCTs: Lefebvre [2] – Cochrane Highly Sensitive Search Strategy for identifying randomized trials in MEDLINE: sensitivity-maximizing version (2008 revision)

| # | Searches |
| --- | --- |
| 1 | Negative-Pressure Wound Therapy/ |
| 2 | (Vacuum/ or Suction/ or Pressure/) and Wound Healing/ |
| 3 | ((vacuum or negative) adj3 (assisted or pressure) adj3 (therap* or dressing* or wound* or closure*)).ti,ab. |
| 4 | or/1-3 |
| 5 | randomized controlled trial.pt. |
| 6 | controlled clinical trial.pt. |
| 7 | randomized.ab. |
| 8 | placebo.ab. |
| 9 | drug therapy.fs. |
| 10 | randomly.ab. |
| 11 | trial.ab. |
| 12 | groups.ab. |
| 13 | or/5-12 |
| 14 | exp animals/ not humans.sh. |
| 15 | 13 not 14 |
| 16 | cochrane database of systematic reviews.jn. |
| 17 | (search or MEDLINE or systematic review).tw. |
| 18 | meta analysis.pt. |
| 19 | or/16-18 |
| 20 | or/15,19 |
| 21 | and/4,20 |
| 22 | 21 not (comment or editorial).pt. |
| 23 | limit 22 to yr="2006-Current" |

PubMed via NLM

- PubMed – as supplied by publisher
- PubMed – in process

PubMed – pubmednotmedline

| **Search** | **Query** |
| --- | --- |
| #1 | Search (vacuum[TIAB] OR negative[TIAB]) AND (assisted[TIAB] OR pressure[TIAB]) AND (therap*[TIAB] OR dressing*[TIAB] OR wound*[TIAB] OR closure*[TIAB]) |
| #2 | Search clinical trial*[TIAB] OR random*[TIAB] OR placebo[TIAB] OR trial[TI] |
| #3 | Search search[TIAB] OR meta analysis[TIAB] OR MEDLINE[TIAB] OR systematic review[TIAB] |
| #4 | Search #2 OR #3 |
| #5 | Search #1 AND #4 |
| #6 | Search #5 NOT Medline[SB] |
| #7 | Search #6 AND 2006:2018[DP] |

Embase via Ovid

Embase 1974 to 2018 July 20

Search filters:

- Systematic Reviews: Wong [1] – High specificity strategy;

RCTs: Wong [1] – Strategy minimizing difference between sensitivity and specificity

| **#** | **Searches** |
| --- | --- |
| 1 | vacuum assisted closure/ |
| 2 | negative pressure wound therapy/ |
| 3 | vacuum assisted closure device/ |
| 4 | (vacuum/ or suction/ or pressure/) and wound healing/ |
| 5 | ((vacuum or negative) adj3 (assisted or pressure) adj3 (therap* or dressing* or wound* or closure*)).ti,ab. |
| 6 | or/1-5 |
| 7 | (random* or double-blind*).tw. |
| 8 | placebo*.mp. |
| 9 | or/7-8 |
| 10 | (meta analysis or systematic review or MEDLINE).tw. |
| 11 | or/9-10 |
| 12 | and/6,11 |
| 13 | 12 not medline.cr. |
| 14 | 13 not (exp animal/ not exp humans/) |
| 15 | 14 not (Conference Abstract or Conference Review or Editorial).pt. |
| 16 | ..l/ 15 yr=2006-Current |

The Cochrane Library via Wiley

- Cochrane Database of Systematic Reviews: Issue 7 of 12, July 2018

Cochrane Central Register of Controlled Trials: Issue 6 of 12, June 2018

| **ID** | **Search** |
| --- | --- |
| #1 | MeSH descriptor: [Negative-Pressure Wound Therapy] this term only |
| #2 | MeSH descriptor: [Vacuum] this term only |
| #3 | MeSH descriptor: [Suction] this term only |
| #4 | MeSH descriptor: [Pressure] this term only |
| #5 | MeSH descriptor: [Wound Healing] this term only |
| #6 | (#2 or #3 or #4) and #5 |
| #7 | ((vacuum or negative) near/3 (assisted or pressure) near/3 (therap* or dressing* or wound* or closure*)):ti,ab |
| #8 | #1 or #6 or #7 |
| #9 | #8 in Cochrane Reviews (Reviews and Protocols) |
| #10 | #8 Publication Year from 2006 to 2018, in Trials |

Health Technology Assessment Database via Centre for Reviews and Dissemination

| **Line** | **Search** |
| --- | --- |
| 1 | MeSH DESCRIPTOR Negative-Pressure Wound Therapy |
| 2 | MeSH DESCRIPTOR Vacuum |
| 3 | MeSH DESCRIPTOR Suction |
| 4 | MeSH DESCRIPTOR Pressure |
| 5 | #2 OR #3 OR #4 |
| 6 | MeSH DESCRIPTOR Wound Healing |
| 7 | #5 AND #6 |
| 8 | ((vacuum or negative) AND (assisted or pressure) AND (therap* or dressing* or wound* or closure*)) |
| 9 | #1 OR #7 OR #8 |
| 10 | (#9) IN HTA FROM 2006 TO 2018 |

**Manufacturers contacted**

1. Asskea GmbH,
2. Atmos Medizin Technik GmbH & Co. KG,
3. Cardinal Health, Inc.,
4. Carilex Medical GmbH,
5. ConvaTec (Germany) GmbH,
6. Cork Medical,
7. Devon Medical Products,
8. Equinox Medical, LLC,
9. Eurosets GmbH,
10. Foryou Medical Electronics Co., Inc.,
11. Healyx Labs,
12. Integrated Healing Technologies,
13. KCI Medizinprodukte GmbH / Acelity,
14. Lohmann & Rauscher GmbH und Co. KG,
15. Medela GmbH & Co. Handels KG,
16. Meditop BV,
17. Mölnlycke Health Care AG,
18. Paul Hartmann AG,
19. Premco Medical Systems, Inc.,
20. Progressive Wound Care,
21. Prospera, Faith CATX, DMLA, LLC.,
22. Smith & Nephew GmbH,
23. Talley Group Limited and
24. Wuhan VSD Medical Science and Technology Co Ltd.

**References**

1. Wong SSL, Wilczynski NL, Haynes RB. Comparison of top-performing search strategies for detecting clinically sound treatment studies and systematic reviews in MEDLINE and EMBASE. J Med Libr Assoc 2006; 94(4): 451-455.

2. Lefebvre C, Manheimer E, Glanville J. Searching for studies [online]. In: Higgings JPT, Green S (Ed). Cochrane handbook for systematic reviews of interventions: version 5.1.0. 03.2011 [Zugriff: 05.09.2018]. URL: <http://handbook-5-1.cochrane.org/chapter_6/6_searching_for_studies.htm>.
