# Supplementary material for: Negative pressure wound therapy in patients with wounds healing by secondary intention: a systematic review and meta-analysis of randomised controlled trials
Source: Syst Rev. 2020 Oct 10;9:238. doi: 10.1186/s13643-020-01476-6 (PMC7548038; doi:10.1186/s13643-020-01476-6)
Supplement: Supplementary file 2 — Additional file 2. Detailed risk of bias for assessments of outcomes (DOCX 49 kb) [file 13643_2020_1476_MOESM2_ESM.docx]

**Additional file 2: Detailed risk of bias for assessments of outcomes**

**Wound closure:**

34 studies reported data on wound closure. Of these only 3 studies had a low risk of bias for this outcome (Ashby 2012, Llanos 2006; SWHSI). All other studies were rated with a high risk of bias.

Table 1: Wound closure: risk of bias at outcome level

| Study | Risk of bias: study level | Blinding of outcome assessment | ITT analysis appropriate | Selective reporting improbable | Absence of other factors potentially causing bias | Risk of bias: outcome level^a^ |
| --- | --- | --- | --- | --- | --- | --- |
| Acosta 2013 | high | n. a. | n. a. | n. a. | n. a. | high |
| Arti 2016 | high | n. a. | n. a. | n. a. | n. a. | high |
| Ashby 2012 | low | yes | yes | yes | yes | low |
| Biter 2014 | high | n. a. | n. a. | n. a. | n. a. | high |
| Braakenburg 2006 | high | n. a. | n. a. | n. a. | n. a. | high |
| *CE/044/PIC* | high | n. a. | n. a. | n. a. | n. a. | high |
| Dalla Paola 2010 S-II | high | n. a. | n. a. | n. a. | n. a. | high |
| *DiaFu* | low | yes | unclear | yes | yes | high |
| Gupta 2013 | high | n. a. | n. a. | n. a. | n. a. | high |
| Hu 2009 | high | n. a. | n. a. | n. a. | n. a. | high |
| Jayakumar 2013 | high | n. a. | n. a. | n. a. | n. a. | high |
| Kakagia 2014 | high | n. a. | n. a. | n. a. | n. a. | high |
| Karatepe 2011 | high | n. a. | n. a. | n. a. | n. a. | high |
| Leclercq 2016 | high | n. a. | n. a. | n. a. | n. a. | high |
| Llanos 2006 | low | yes | yes | yes | yes | low |
| Moisidis 2004 | high | n. a. | n. a. | n. a. | n. a. | high |
| Mouës 2004 | high | n. a. | n. a. | n. a. | n. a. | high |
| Nain 2011 | high | n. a. | n. a. | n. a. | n. a. | high |
| Novinščak 2010 | high | n. a. | n. a. | n. a. | n. a. | high |
| Perez 2010 | high | n. a. | n. a. | n. a. | n. a. | high |
| Saaiq 2010 | high | n. a. | n. a. | n. a. | n. a. | high |
| Shen 2013 | high | n. a. | n. a. | n. a. | n. a. | high |
| Sibin 2017 | high | n. a. | n. a. | n. a. | n. a. | high |
| SWHSI | low | no | yes | yes | yes | low |
| *VAC 2001-03* | high | n. a. | n. a. | n. a. | n. a. | high |
| VAC 2001-06 | high | n. a. | n. a. | n. a. | n. a. | high |
| VAC 2001-07 | low | no | unclear | yes | yes | high |
| VAC 2001-08 | high | n. a. | n. a. | n. a. | n. a. | high |
| *VAC 2002-09* | high | n. a. | n. a. | n. a. | n. a. | high |
| *VAC 2002-10* | high | n. a. | n. a. | n. a. | n. a. | high |
| Virani 2016 | high | n. a. | n. a. | n. a. | n. a. | high |
| Vuerstaek 2006 | low | no | unclear | yes | yes | high |
| WOLLF | low | yes | no | yes | yes | high |
| Xu 2015 | high | n. a. | n. a. | n. a. | n. a. | high |
| *Study title in italics*: unpublished study  ITT: intention to treat; n. a.: not applicable  a: A high risk of bias at study level resulted in a high risk of bias at outcome level. Thus, no further evaluations of the remaining items were performed. | | | | | | |

**Adverse events:**

41 studies reported data on wound complications and treatment complications. The risk of bias for the reported outcomes was low for only one study (Llanos 2006). All other studies were rated with a high risk of bias for all outcomes.

Table 2: Adverse events: risk of bias at outcome level

| **Study** | Risk of bias: study level | Blinding of outcome assessment | ITT analysis appropriate | Selective reporting improbable | Absence of other factors potentially causing bias | Risk of bias: outcome level^a^ |
| --- | --- | --- | --- | --- | --- | --- |
| Acosta 2013 | high | n. a. | n. a. | n. a. | n. a. | high |
| Arti 2016 | high | n. a. | n. a. | n. a. | n. a. | high |
| Ashby 2012 | low | unclear | yes | yes | yes | high |
| Bee 2008 | high | n. a. | n. a. | n. a. | n. a. | high |
| Biter 2014 | high | n. a. | n. a. | n. a. | n. a. | high |
| Braakenburg 2006 | high | n. a. | n. a. | n. a. | n. a. | high |
| *CE/044/PIC* | high | n. a. | n. a. | n. a. | n. a. | high |
| Chiang 2017 | high | n. a. | n. a. | n. a. | n. a. | high |
| Dalla Paola 2010 S-II | high | n. a. | n. a. | n. a. | n. a. | high |
| De Laat 2011 | high | n. a. | n. a. | n. a. | n. a. | high |
| *DiaFu* | low | no | unclear | yes | yes | high |
| Gupta 2013 | high | n. a. | n. a. | n. a. | n. a. | high |
| Hu 2009 | high | n. a. | n. a. | n. a. | n. a. | high |
| Jayakumar 2013 | high | n. a. | n. a. | n. a. | n. a. | high |
| Kakagia 2014 | high | n. a. | n. a. | n. a. | n. a. | high |
| Liao 2012 | high | n. a. | n. a. | n. a. | n. a. | high |
| Llanos 2006 | low | unclear | yes | yes | yes | low |
| Mody 2008 | high | n. a. | n. a. | n. a. | n. a. | high |
| Mohsin 2017 | high | n. a. | n. a. | n. a. | n. a. | high |
| Moisidis 2004 | high | n. a. | n. a. | n. a. | n. a. | high |
| Mouës 2004 | high | n. a. | n. a. | n. a. | n. a. | high |
| Nain 2011 | high | n. a. | n. a. | n. a. | n. a. | high |
| Perez 2010 | high | n. a. | n. a. | n. a. | n. a. | high |
| Rencüzoğulları 2015 | high | n. a. | n. a. | n. a. | n. a. | high |
| Saaiq 2010 | high | n. a. | n. a. | n. a. | n. a. | high |
| Sibin 2017 | high | n. a. | n. a. | n. a. | n. a. | high |
| Sinha 2013 | high | n. a. | n. a. | n. a. | n. a. | high |
| SWHSI | low | no | unclear | yes | yes | high |
| TOPSKIN | high | n. a. | n. a. | n. a. | n. a. | high |
| *VAC 2001-01* | high | n. a. | n. a. | n. a. | n. a. | high |
| *VAC 2001-02* | high | n. a. | n. a. | n. a. | n. a. | high |
| *VAC 2001-03* | high | n. a. | n. a. | n. a. | n. a. | high |
| VAC 2001-06 | high | n. a. | n. a. | n. a. | n. a. | high |
| VAC 2001-07 | low | no | unclear | yes | yes | high |
| VAC 2001-08 | high | n. a. | n. a. | n. a. | n. a. | high |
| *VAC 2002-09* | high | n. a. | n. a. | n. a. | n. a. | high |
| *VAC 2002-10* | high | n. a. | n. a. | n. a. | n. a. | high |
| Virani 2016 | high | n. a. | n. a. | n. a. | n. a. | high |
| Vuerstaek 2006 | low | no | unclear | yes | yes | high |
| WOLLF | low | no | yes / no^b^ | yes / unclear^c^ | yes | high |
| Xu 2015 | high | n. a. | n. a. | n. a. | n. a. | high |
| *Study title in italics*: unpublished study  ITT: intention to treat; n. a.: not applicable; (S)AE: (serious) adverse event  a: A high risk of bias at study level resulted in a high risk of bias at outcome level. Thus, no further evaluations of the remaining items were performed.  b: “Yes” refers to the outcome “infection”; “no” refers to “further surgery for wound closure” and “re-intervention”.  c: “Yes” refers to the outcomes “further surgery for wound closure” and “re-intervention”; “unclear” refers to “infection”. | | | | | | |

**Length of hospital stay and/or readmission to hospital:**

17 studies reported data on length of hospital stay and/or readmission to hospital. The risk of bias for this outcome was low for only one study (Llanos 2006). All other studies were rated with a high risk of bias.

Table 3: Length of hospital stay and/or readmission to hospital

| Study | Risk of bias: study level | Blinding of outcome assessment | ITT analysis appropriate | Selective reporting improbable | Absence of other factors potentially causing bias | Risk of bias: outcome level^a^ |
| --- | --- | --- | --- | --- | --- | --- |
| Acosta 2013 | high | n. a. | n. a. | n. a. | n. a. | high |
| CE/044/PIC | high | n. a. | n. a. | n. a. | n. a. | high |
| De Laat 2011 | high | n. a. | n. a. | n. a. | n. a. | high |
| Gupta 2013 | high | n. a. | n. a. | n. a. | n. a. | high |
| Huang 2006 | high | n. a. | n. a. | n. a. | n. a. | high |
| Jayakumar 2013 | high | n. a. | n. a. | n. a. | n. a. | high |
| Liao 2012 | high | n. a. | n. a. | n. a. | n. a. | high |
| Llanos 2006 | low | unclear | yes | yes | yes | low |
| Rencüzoğulları 2015 | high | n. a. | n. a. | n. a. | n. a. | high |
| Saaiq 2010 | high | n. a. | n. a. | n. a. | n. a. | high |
| Sibin 2017 | high | n. a. | n. a. | n. a. | n. a. | high |
| SWHSI | low | no | unclear | yes | yes | high |
| TOPSKIN | high | n. a. | n. a. | n. a. | n. a. | high |
| VAC 2001-06 | high | n. a. | n. a. | n. a. | n. a. | high |
| VAC 2001-07 | low | no | unclear | yes | yes | high |
| Vuerstaek 2006 | low | no | unclear | yes | yes | high |
| Xu 2015 | high | n. a. | n. a. | n. a. | n. a. | high |
| *Study title in italics*: unpublished study  ITT: intention to treat; n. a.: not applicable  a: A high risk of bias at study level resulted in a high risk of bias at outcome level. Thus, no further evaluations of the remaining items were performed. | | | | | | |

**Mortality:**

18 studies reported data on death. The risk of bias for this outcome was low for only one study (Ashby 2012). All other studies were rated with a high risk of bias.

Table 4: Mortality: risk of bias at outcome level

| Study | Risk of bias: study level | Blinding of outcome assessment | ITT analysis appropriate | Selective reporting improbable | Absence of other factors potentially causing bias | Risk of bias: outcome level^a^ |
| --- | --- | --- | --- | --- | --- | --- |
| Acosta 2013 | high | n. a. | n. a. | n. a. | n. a. | high |
| Ashby 2012 | low | no | yes | yes | yes | low |
| Bee 2008 | high | n. a. | n. a. | n. a. | n. a. | high |
| Braakenburg 2006 | high | n. a. | n. a. | n. a. | n. a. | high |
| Correa 2016 | high | n. a. | n. a. | n. a. | n. a. | high |
| DiaFu | high | n. a. | n. a. | n. a. | n. a. | high |
| Huang 2006 | high | n. a. | n. a. | n. a. | n. a. | high |
| Mouës 2004 | high | n. a. | n. a. | n. a. | n. a. | high |
| Rencüzoğulları 2015 | high | n. a. | n. a. | n. a. | n. a. | high |
| Saaiq 2010 | high | n. a. | n. a. | n. a. | n. a. | high |
| *VAC 2001-01* | high | n. a. | n. a. | n. a. | n. a. | high |
| VAC 2001-07 | low | no | unclear | yes | no^b^ | high |
| VAC 2001-08 | high | n. a. | n. a. | n. a. | n. a. | high |
| *VAC 2002-09* | high | n. a. | n. a. | n. a. | n. a. | high |
| *VAC 2002-10* | high | n. a. | n. a. | n. a. | n. a. | high |
| Vuerstaek 2006 | low | no | yes | yes | no^b^ | high |
| WOLLF | low | no | yes | yes | no^b^ | high |
| Xu 2015 | high | n. a. | n. a. | n. a. | n. a. | high |
| *Study title in italics*: unpublished study  ITT: intention to treat; n. a.: not applicable  a: A high risk of bias at study level resulted in a high risk of bias at outcome level. Thus, no further evaluations of the remaining items were performed.  b: Mortality was not recorded systematically in this study. | | | | | | |

**Amputation:**

10 studies reported data on amputations. The risk of bias for this outcome was low for only one study (SWHSI). All other studies were rated with a high risk of bias.

Table 5: Amputation: risk of bias at outcome level

| Study | Risk of bias: study level | Blinding of outcome assessment | ITT analysis appropriate | Selective reporting improbable | Absence of other factors potentially causing bias | Risk of bias: outcome level^a^ |
| --- | --- | --- | --- | --- | --- | --- |
| Acosta 2013 | high | n. a. | n. a. | n. a. | n. a. | high |
| Braakenburg 2006 | high | n. a. | n. a. | n. a. | n. a. | high |
| Dalla Paola 2010 S-II | high | n. a. | n. a. | n. a. | n. a. | high |
| *DiaFu* | low | unclear | unclear | yes | yes | high |
| Hu 2009 | high | n. a. | n. a. | n. a. | n. a. | high |
| Huang 2006 | high | n. a. | n. a. | n. a. | n. a. | high |
| Mody 2008 | high | n. a. | n. a. | n. a. | n. a. | high |
| SWHSI | low | no | yes | yes | yes | low |
| VAC 2001-06 | high | n. a. | n. a. | n. a. | n. a. | high |
| WOLLF | low | no | no | yes | yes | high |
| *Study title in italics*: unpublished study  ITT: intention to treat; n. a.: not applicable  a: A high risk of bias at study level resulted in a high risk of bias at outcome level. Thus, no further evaluation of the remaining items was performed. | | | | | | |

**Pain:**

10 studies reported data on pain. All studies were rated with a high risk of bias for this outcome.

Table 6: Pain: risk of bias at outcome level

| Study | Risk of bias: study level | Blinding of outcome assessment | ITT analysis appropriate | Selective reporting improbable | Absence of other factors potentially causing bias | Risk of bias: outcome level^a^ |
| --- | --- | --- | --- | --- | --- | --- |
| Ashby 2012 | low | no | yes | yes | yes | high |
| Banasiewicz 2013 | high | n. a. | n. a. | n. a. | n. a. | high |
| Biter 2014 | high | n. a. | n. a. | n. a. | n. a. | high |
| *CE/044/PIC* | high | n. a. | n. a. | n. a. | n. a. | high |
| *DiaFu* | low | no | yes | yes | yes | high |
| Mody 2008 | high | n. a. | n. a. | n. a. | n. a. | high |
| SWHSI | low | no | no | yes | yes | high |
| TOPSKIN | high | n. a. | n. a. | n. a. | n. a. | high |
| Vuerstaek 2006 | low | no | unclear | yes | yes | high |
| WOLLF | low | no | no | yes | yes | high |
| *Study title in italics*: unpublished study  ITT: intention to treat; n. a.: not applicable.  a: A high risk of bias at study level resulted in a high risk of bias at outcome level. Thus, no further evaluations of the remaining items were performed. | | | | | | |

**Health-related quality of life:**

2 studies reported data on quality of life. Both were rated with a high risk of bias for this outcome.

Table 7: Health-related quality of life: risk of bias at outcome level

| Study | Risk of bias: study level | Blinding of outcome assessment | ITT analysis appropriate | Selective reporting improbable | Absence of other factors potentially causing bias | Risk of bias: outcome level^a^ |
| --- | --- | --- | --- | --- | --- | --- |
| SWHSI | low | no | no | yes | yes | high |
| WOLLF | low | no | no | yes | yes | high |
| ITT: intention to treat; n. a.: not applicable  a: A high risk of bias at study level resulted in a high risk of bias at outcome level. Thus, no further evaluations of the remaining items were performed. | | | | | | |

**Physical function:**

3 studies reported data on function. All were rated with a high risk of bias for this outcome.

Table 8: Physical function: risk of bias at outcome level

| Study | Risk of bias: study level | Blinding of outcome assessment | ITT analysis appropriate | Selective reporting improbable | Absence of other factors potentially causing bias | Risk of bias: outcome level^a^ |
| --- | --- | --- | --- | --- | --- | --- |
| Banasiewicz 2013 | high | n. a. | n. a. | n. a. | n. a. | high |
| Biter 2014 | high | n. a. | n. a. | n. a. | n. a. | high |
| WOLLF | low | no | no | yes | yes | high |
| ITT: intention to treat; n. a.: not applicable  a: A high risk of bias at study level resulted in a high risk of bias at outcome level. Thus, no further evaluations of the remaining items were performed. | | | | | | |
