# Supplementary material for: Negative pressure wound therapy in patients with wounds healing by secondary intention: a systematic review and meta-analysis of randomised controlled trials
Source: Syst Rev. 2020 Oct 10;9:238. doi: 10.1186/s13643-020-01476-6 (PMC7548038; doi:10.1186/s13643-020-01476-6)
Supplement: Supplementary file 3 — Additional file 3. Forest plots not presented in the manuscript (DOCX 62 kb) [file 13643_2020_1476_MOESM3_ESM.docx]

**Additional file 3: Forest plots not presented in the manuscript**

Fig. 1: Forest plot of wound healing and / or surgical wound closure with 95% PI, NPWT vs. SWT.
Abbreviations: CI = confidence interval, n = number of events, N = number of patients, NPWT = negative pressure wound therapy, OR = odds ratio, PI = prediction interval, SWT = standard wound therapy.

Fig. 2: Forest plot of time to wound healing and / or surgical wound closure (in days) with 95% PI, NPWT vs. SWT.
Abbreviations: CI = confidence interval, n = number of patients, NPWT = negative pressure wound therapy, PI = prediction interval, SD = standard deviation, SWT = standard wound therapy.

Fig. 3: Forest plot of AEs: Re-intervention with overall effect estimation, NPWT vs. SWT.
Abbreviations: AE = adverse event, CI = confidence interval, n = number of events, N = number of patients, NPWT = negative pressure wound therapy, OR = odds ratio, SWT = standard wound therapy.

Fig. 4: Forest plot of AEs: Additional measures required for direct wound closure with overall effect estimation, NPWT vs. SWT.
Abbreviations: AE = adverse event, CI = confidence interval, n = number of events, N = number of patients, NPWT = negative pressure wound therapy, OR = odds ratio, SWT = standard wound therapy.

Fig. 5: Forest plot of overall rate of serious adverse events with overall effect estimation, NPWT vs. SWT.
Abbreviations: CI = confidence interval, n = number of events, N = number of patients, NPWT = negative pressure wound therapy, OR = odds ratio, SWT = standard wound therapy.

Fig. 6: Forest plot of AEs: Infection with 95% PI, NPWT vs. SWT.
Abbreviations: AE = adverse event, CI = confidence interval, n = number of events, N = number of patients, NPWT = negative pressure wound therapy, OR = odds ratio, PI = prediction interval, SWT = standard wound therapy.

Fig. 7: Forest plot of separate reported SAEs - Discontinuation of the study due to adverse events with 95% PI, NPWT vs. SWT.
Abbreviations: CI = confidence interval, n = number of events, N = number of patients, NPWT = negative pressure wound therapy, OR = odds ratio, PI = prediction interval, SAE = serious adverse event, SWT = standard wound therapy.

Fig. 8: Forest plot of AEs: Bleeding without overall effect estimation, NPWT vs. SWT.
Abbreviations: AE = adverse event, CI = confidence interval, n = number of events, N = number of patients, NPWT = negative pressure wound therapy, OR = odds ratio, SWT = standard wound therapy, w ds = with dermal substitute, w/o ds = without dermal substitute.

Fig. 9: Forest plot of readmission with overall effect estimation, NPWT vs. SWT.
Abbreviations: CI = confidence interval, n = number of events, N = number of patients, NPWT = negative pressure wound therapy, OR = odds ratio, SWT = standard wound therapy.

Fig. 10: Forest plot of mortality with overall effect estimation, NPWT vs. SWT.
Abbreviations: CI = confidence interval, n = number of events, N = number of patients, NPWT = negative pressure wound therapy, OR = odds ratio, SWT = standard wound therapy.

Fig. 11: Forest plot of amputation with overall effect estimation, NPWT vs. SWT.
Abbreviations: CI = confidence interval, n = number of events, N = number of patients, NPWT = negative pressure wound therapy, OR = odds ratio, SWT = standard wound therapy.

Fig. 12: Forest plot of pain – continuous with overall effect estimation, NPWT vs. SWT.
Abbreviations: CI = confidence interval, n = number of patients, NPWT = negative pressure wound therapy, SD = standard deviation, SWT = standard wound therapy.

Fig. 13: Forest plot of pain – dichotomous without overall effect estimation, NPWT vs. SWT.
Abbreviations: CI = confidence interval, n = number of events, N = number of patients, NPWT = negative pressure wound therapy, OR = odds ratio, SWT = standard wound therapy.

| eTable: 1: Pain on application and removal of dressing (continuous) | | | | | | | | |
| --- | --- | --- | --- | --- | --- | --- | --- | --- |
| Study  Instrument / [Range] | Time of evaluation | NPWT | |  | SWT | |  | NPWT vs. SWT |
|  |  | N | Mean (SD) |  | N | Mean (SD) |  | MD [95% CI]; p-value |
| SWHSI | 2 weeks | 13 | 22.3 (25.5) |  | 15 | 22.6 (24.5) |  | −0.30 [−19.75; 19.15]; 0.975^b^ |
| VAS / [n. r.]^a^ |  |  |  |  |  |  |  |  |
| CI: confidence interval; MD: mean difference; N: number of analysed patients; NPWT: negative pressure wound therapy; n. r.: not reported; SD: standard deviation; SWT: standard wound therapy; VAS: visual analogue scale; vs.: versus  a: According to the information in the publication, a scale of 0 –10 was used, but the results indicate a scale of 0–100.  b: IQWiG’s own calculation of MD, 95 % CI and p-value (t-test) | | | | | | | | |

| eTable 2: Pain on application and removal of dressing (dichotomous) | | | | | | | | |
| --- | --- | --- | --- | --- | --- | --- | --- | --- |
| Study | Time of evaluation | NPWT | |  | SWT | |  | NPWT vs. SWT |
|  |  | N | n (%) |  | N | n (%) |  | OR [95% CI]; p‑value^a^ |
| Patients with pain (on application of dressing)^b^ | | | | | | | | |
| *CE/044/PIC* | week 0 | 30 | 1 (3.3) |  | 30 | 4 (13.3) |  | 0.22 [0.02; 2.14]; 0.219 |
|  | week 1 | 28 | 1 (3.6) |  | 30 | 0 (0) |  | 3.33 [0.13; 85.11]; 0.359 |
|  | week 2 | 26 | 0 (0) |  | 29 | 3 (10.3) |  | 0.14 [0.01; 2.90]; 0.107 |
|  | week 3 | 23 | 1 (4.3) |  | 27 | 2 (7.4) |  | 0.57 [0.05; 6.70]; 0.714 |
|  | week 4 | 19 | 1 (5.3) |  | 26 | 2 (7.7) |  | 0.67 [0.06; 7.94]; 0.824 |
| Patients with pain (on removal of dressing)^b^ | | | | | | | | |
| *CE/044/PIC* | week 1 | 28 | 4 (14.3)^c^ |  | 31 | 3 (9.7)^c^ |  | 1.56 [0.32; 7.65]; 0.636 |
|  | week 2 | 26 | 3 (11.5)^c^ |  | 29 | 3 (10.3) |  | 1.13 [0.21; 6.16]; 0.949 |
|  | week 3 | 23 | 3 (13.0) |  | 27 | 2 (7.4)^c^ |  | 1.88 [0.29; 12.33]; 0.611 |
|  | week 4 | 19 | 1 (5.3) |  | 26 | 4 (15.4) |  | 0.31 [0.03; 2.98]; 0.350 |
| *Study title in italics*: unpublished study  CI: confidence interval; n: number of patients with an event; N: number of analysed patients; NPWT: negative pressure wound therapy; OR: odds ratio; SWT: standard wound therapy; vs.: versus  a:IQWiG’s own calculation of OR, 95% CI (asymptotic) and p-value (unconditional exact test, CSZ method according to [1]).  b: Only patients with at least moderate pain (moderate and severe) are considered. Patients with slight or no pain are not considered.  c: IQWiG’s own calculation. | | | | | | | | |

Fig. 14: Forest plot of health-related quality of life (SF-12 MCS) with overall effect estimation, NPWT vs. SWT.
Abbreviations: CI = confidence interval, MCS = Mental Health Composite Scale, n = number of patients, NPWT = negative pressure wound therapy, SD = standard deviation, SF = Short Form, SWT = standard wound therapy.

Fig. 15: Forest plot of health-related quality of life (SF-12 PCS) without overall effect estimation, NPWT vs. SWT.
Abbreviations: CI = confidence interval, n = number of patients, NPWT = negative pressure wound therapy, PCS = Physical Composite Scale, SD = standard deviation, SF = Short Form, SWT = standard wound therapy.

Fig. 16: Forest plot of physical function without overall effect estimation, NPWT vs. SWT.
Abbreviations: CI = confidence interval, n = number of patients, NPWT = negative pressure wound therapy, SD = standard deviation, SWT = standard wound therapy.

| eTable 3: Physical function | | | | | | | | |
| --- | --- | --- | --- | --- | --- | --- | --- | --- |
| Study | Time of evaluation | NPWT | |  | SWT | |  | NPWT vs. SWT |
|  |  | N | Mean (SD) |  | N | Mean (SD) |  | MD [95% CI]; p-value |
| Disability Rating Index [0–100]^a^ | | | | | | | | |
| WOLLF | 3 months | 166 | 64.3 (22.3) |  | 188 | 65.6 (20.1) |  | 0.7 [−3.7; 5.0]; 0.76^b^ |
|  | 6 months | 154 | 53.2 (23.8) |  | 175 | 50.3 (24.1) |  | −3.5 [−8.4; 1.5]; 0.17^b^ |
|  | 12 months | 179 | 45.5 (28.0) |  | 195 | 42.4 (24.2) |  | −3.9 [−8.9; 1.2]; 0.13^b^ |
| ANOVA: analysis of variance; CI: confidence interval; MD: mean value difference; N: number of analysed patients; NPWT: negative pressure wound therapy; SWT: standard wound therapy; SD: standard deviation; vs.: versus  a: Lower values correspond to an improvement.  b: Mixed-model with treatment, age group, sex, Preinjury Score to baseline, and classification of the wound according to Gustilo-Anderson as fixed effects and study centre as random effect. The p-value belongs to the ANOVA F-Test. | | | | | | | | |

References

1. Martin Andrés A, Silva Mato A. Choosing the optimal unconditioned test for comparing two independent proportions. Comput Stat Data Anal 1994; 17(5): 555-574.
