# Supplementary material for: Negative pressure wound therapy in patients with wounds healing by secondary intention: a systematic review and meta-analysis of randomised controlled trials
Source: Syst Rev. 2020 Oct 10;9:238. doi: 10.1186/s13643-020-01476-6 (PMC7548038; doi:10.1186/s13643-020-01476-6)
Supplement: Supplementary file 4 — Additional file 4. Overview of key findings according to GRADE (DOCX 18 kb) [file 13643_2020_1476_MOESM4_ESM.docx]

#### Additional file 4: Overview of key findings according to GRADE methods

| **Summary of findings:** | | | | | | | | | | | | |
| --- | --- | --- | --- | --- | --- | --- | --- | --- | --- | --- | --- | --- |
| **NPWT compared to standard wound therapy in patients with wounds healing by secondary intention** | | | | | | | | | | | | |
| **Patient or population**: Patients with wounds healing by secondary intention  **Setting**: Any | | | **Intervention**: NPWT  **Comparison**: Conventional wound therapy | | | | | | | | | |
| **Outcomes** | | **Anticipated absolute effects^*^** (95% CI) | | | | **Relative effect (95% CI)** | **№ of participants  (studies)** | | **Certainty of the evidence (GRADE)** | | **Comments** | |
|  |  | **Risk with standard wound therapy** | | | **Risk with  NPWT** |  |  |  |  |  |  |  |
| Mortality | 8 per 100 | | | **9 per 100** (6 to 12) | | **OR 1.20** (0.84 to 1.70) | | 2287 (18 RCTs) | | ⨁⨁⨁◯ MODERATE ^a^ | | NPWT likely results in little to no difference in mortality. |
| Wound healing | 34 per 100 | | | **44 per 100** (37 to 52) | | **OR 1.56** (1.15 to 2.13) | | 1094 (14 RCTs) | | ⨁⨁⨁◯ MODERATE ^a^ | | NPWT likely increases wound healing slightly. |
| Serious adverse events | 38 per 100 | | | **39 per 100** (32 to 46) | | **OR 1.02** (0.76 to 1.37) | | 1616 (12 RCTs) | | ⨁⨁⨁◯ MODERATE ^a,b^ | | NPWT likely results in little to no difference in serious adverse events. |
| Amputation | 9 per 100 | | | **8 per 100** (5 to 13) | | **OR 0.89** (0.55 to 1.43) | | 1255 (10 RCTs) | | ⨁⨁⨁◯ MODERATE ^a^ | | NPWT likely results in little to no difference in amputation. |
| Pain | The mean pain ranged from **1.5 to 3.2** (on 0-to-10 scale) | | | - | | Hedges' g **0.16 lower** (0.53 lower to 0.21 higher) | | 587 (6 RCTs) | | ⨁⨁⨁◯ MODERATE ^a^ | | NPWT likely results in little to no difference in pain. |
| Length of hospital stay | The mean length of hospital stay ranged from **12 to 45** days | | | mean **4.8 days lower** (1.8 lower to 7.8 lower) | | - | | 560 (10 RCTs) | | ⨁⨁⨁◯ MODERATE ^a^ | | NPWT likely reduces length of hospital stay slightly. |
| Health-related quality of life | The effect of NPWT on the Physical Composite Scale of the SF-36 was heterogeneous. No effect was found on the Mental Health Composite Scale of the SF-36 (Hedges' g 0.01; 95%-CI: −0.20 to 0.22). | | | | | | | 357 (2 RCTs) | | ⨁⨁◯◯ LOW ^a,c^ | | NPWT may result in little to no difference in health-related quality of life. |
| ***The risk in the intervention group** (and its 95% confidence interval) is based on the assumed risk in the comparison group and the **relative effect** of the intervention (and its 95% CI).   **CI:** Confidence interval; **OR:** Odds ratio ; NPWT: Negative pressure wound therapy; RCTs: Randomized controlled trials; SF‑36: Short form 36 | | | | | | | | | | | | |

#### Explanations

a. Downgraded by one level for probable publication bias, as 24% of results data were inaccessible.

b. Additional analyses were performed on specific adverse events (e.g. wound infection or bleeding), but no differences were detectable.

c. Downgraded by one level due to imprecision, as results from only 2 studies were available on this outcome. In addition, results were heterogeneous.
