# Supplementary material for: Negative pressure wound therapy in patients with wounds healing by secondary intention: a systematic review and meta-analysis of randomised controlled trials
Source: Syst Rev. 2020 Oct 10;9:238. doi: 10.1186/s13643-020-01476-6 (PMC7548038; doi:10.1186/s13643-020-01476-6)
Supplement: Supplementary file 5 — Additional file 5. List of previous systematic reviews (DOCX 18 kb) [file 13643_2020_1476_MOESM5_ESM.docx]

**Additional file 5: List of previous systematic reviews**

1. Anghel EL, Kim PJ. Negative-pressure wound therapy: a comprehensive review of the evidence. Plast Reconstr Surg 2016; 138(Suppl 3): 129S-137S.

2. Atema JJ, Gans SL, Boermeester MA. Systematic review and meta-analysis of the open abdomen and temporary abdominal closure techniques in non-trauma patients. World J Surg 2015; 39(4): 912-925.

3. Brassard J, Tardif M. Vers une utilisation optimale de la thérapie par pression négative pour le traitement des plaies complexes: rapport d’évaluation des technologies de la santé. Québec: Institut national d'excellence en sante et en services sociaux; 2015. URL: http://www.crd.york.ac.uk/CRDWeb/ShowRecord.asp?ID=32015001023.

4. Canadian Agency for Drugs and Technologies in Health. Negative pressure wound therapy for managing diabetic foot ulcers: a review of the clinical effectiveness, cost-effectiveness, and guidelines [online]. 28.08.2014 [Accessed: 14.11.2017]. URL: https://www.cadth.ca/media/pdf/htis/dec-2014/RC0579-001%20Diabetic%20Foot%20Ulcers%20Final.pdf.

5. Chiara O, Cimbanassi S, Biffl W, Leppaniemi A, Henry S, Scalea TM et al. International consensus conference on open abdomen in trauma. J Trauma Acute Care Surg 2016; 80(1): 173-183.

6. Cirocchi R, Birindelli A, Biffl WL, Mutafchiyski V, Popivanov G, Chiara O et al. What is the effectiveness of the negative pressure wound therapy (NPWT) in patients treated with open abdomen technique? A systematic review and meta-analysis. J Trauma Acute Care Surg 2016; 81(3): 575-584.

7. Cristaudo A, Jennings S, Gunnarsson R, DeCosta A. Complications and mortality associated with temporary abdominal closure techniques: a systematic review and meta-analysis. Am Surg 2017; 83(2): 191-216.

8. Dumville JC, Hinchliffe RJ, Cullum N, Game F, Stubbs N, Sweeting M et al. Negative pressure wound therapy for treating foot wounds in people with diabetes mellitus. Cochrane Database Syst Rev 2013; (1): CD010318.

9. Dumville JC, Land L, Evans D, Peinemann F. Negative pressure wound therapy for treating leg ulcers. Cochrane Database Syst Rev 2015; (7): CD011354.

10. Dumville JC, Munson C, Christie J. Negative pressure wound therapy for partial-thickness burns. Cochrane Database Syst Rev 2014; (12): CD006215.

11. Dumville JC, Owens GL, Crosbie EJ, Peinemann F, Liu Z. Negative pressure wound therapy for treating surgical wounds healing by secondary intention. Cochrane Database Syst Rev 2015; (6): CD011278.

12. Dumville JC, Webster J, Evans D, Land L. Negative pressure wound therapy for treating pressure ulcers. Cochrane Database Syst Rev 2015; (5): CD011334.

13. Game FL, Apelqvist J, Attinger C, Hartemann A, Hinchliffe RJ, Londahl M et al. Effectiveness of interventions to enhance healing of chronic ulcers of the foot in diabetes: a systematic review. Diabetes Metab Res Rev 2016; 32(Suppl 1): 154-168.

14. Iheozor-Ejiofor Z, Newton K, Dumville JC, Costa ML, Norman G, Bruce J. Negative pressure wound therapy for open traumatic wounds. Cochrane Database Syst Rev 2018; (7): CD012522.

15. Janssen AHJ, Mommers EHH, Notter J, De Vries Reilingh TS, Wegdam JA. Negative pressure wound therapy versus standard wound care on quality of life: a systematic review. J Wound Care 2016; 25(3): 154, 156-159.

16. Liu S, He CZ, Cai YT, Xing QP, Guo YZ, Chen ZL et al. Evaluation of negative-pressure wound therapy for patients with diabetic foot ulcers: systematic review and meta-analysis. Ther Clin Risk Manag 2017; 13: 533-544.

17. Liu X, Zhang H, Cen S, Huang F. Negative pressure wound therapy versus conventional wound dressings in treatment of open fractures: a systematic review and meta-analysis. Int J Surg 2018; 53: 72-79.

18. Malaysian Health Technology Assessment. Disposable negative pressure wound therapy: 026/2013 [online]. [Accessed: 14.11.2017]. URL: http://www.moh.gov.my/index.php/database_stores/attach_download/347/229.

19. National Institute for Health and Care Excellence, Internal Clinical Guidelines team. Diabetic foot problems: prevention and management [online]. 05.2016 [Accessed: 05.07.2017]. (NICE clinical guidelines; Band 19). URL: https://www.nice.org.uk/guidance/ng19/evidence/full-guidance-pdf-15672915543.

20. Nelson EA, Adderley U. Venous leg ulcers. BMJ Clin Evid 2016; 2016: pii: 1902.

21. Pan A, De Angelis G, Nicastri E, Sganga G, Tacconelli E. Topical negative pressure to treat surgical site infections, with a focus on post-sternotomy infections: a systematic review and meta-analysis. Infection 2013; 41(6): 1129-1135.

22. Quecedo L, Del Llano J. Systematic review of studies on efficacy of negative pressure therapies in complex diabetic foot wounds [Spanisch]. Pharmacoecon Span Res Artic 2013; 10(2): 53-59.

23. Reddy M. Pressure ulcers: treatment. BMJ Clin Evid 2015; 2015: pii: 1901.

24. Rhee SM, Valle MF, Wilson LM, Lazarus G, Zenilman JM, Robinson KA. Negative pressure wound therapy technologies for chronic wound care in the home setting: technology assessment report [online]. 15.09.2014 [Accessed: 21.11.2017]. URL: https://www.ncbi.nlm.nih.gov/books/NBK285361/pdf/Bookshelf_NBK285361.pdf.

25. Sharrock AE, Barker T, Yuen HM, Rickard R, Tai N. Management and closure of the open abdomen after damage control laparotomy for trauma: a systematic review and meta-analysis. Injury 2016; 47(2): 296-306.

26. Tansarli GS, Vardakas KZ, Stratoulias C, Peppas G, Kapaskelis A, Falagas ME. Vacuum-assisted closure versus closure without vacuum assistance for preventing surgical site infections and infections of chronic wounds: a meta-analysis of randomized controlled trials. Surg Infect (Larchmt) 2014; 15(4): 363-367.

27. Walker M, Kralik D, Porritt K. Fasciotomy wounds associated with acute compartment syndrome: a systematic review of effective treatment. JBI Database System Rev Implement Rep 2014; 12(1): 101-175.

28. Wang R, Feng Y, Di B. Comparisons of negative pressure wound therapy and ultrasonic debridement for diabetic foot ulcers: a network meta-analysis. Int J Clin Exp Med 2015; 8(8): 12548-12556.

29. Yin Y, Zhang R, Li S, Guo J, Hou Z, Zhang Y. Negative-pressure therapy versus conventional therapy on split-thickness skin graft: a systematic review and meta-analysis. Int J Surg 2018; 50: 43-48.

30. Zhang J, Hu ZC, Chen D, Guo D, Zhu JY, Tang B. Effectiveness and safety of negative-pressure wound therapy for diabetic foot ulcers: a meta-analysis. Plast Reconstr Surg 2014; 134(1): 141-151.
